# Chemogenetic Activation of Melanopsin Retinal Ganglion Cells Induces Signatures of Arousal and/or Anxiety in Mice

**DOI:** 10.1016/j.cub.2016.06.057

**Published:** 2016-09-12

**Authors:** Nina Milosavljevic, Jasmina Cehajic-Kapetanovic, Christopher A. Procyk, Robert J. Lucas

**Affiliations:** 1 Faculty of Life Sciences, University of Manchester, Oxford Road, Manchester M13 9PT, UK; 2 Centre for Ophthalmology and Vision Sciences, Institute of Human Development, University of Manchester, Manchester M13 9PT, UK

## Abstract

Functional imaging and psychometric assessments indicate that bright light can enhance mood, attention, and cognitive performance in humans. Indirect evidence links these events to light detection by intrinsically photosensitive melanopsin-expressing retinal ganglion cells (mRGCs) [1, 2, 3, 4, 5, 6, 7, 8, 9]. However, there is currently no direct demonstration that mRGCs can have such an immediate effect on mood or behavioral state in any species. We addressed this deficit by using chemogenetics to selectively activate mRGCs, simulating the excitatory effects of bright light on this cell type in dark-housed mice. This specific manipulation evoked circadian phase resetting and pupil constriction (known consequences of mRGC activation). It also induced c-Fos (a marker of neuronal activation) in multiple nuclei in the hypothalamus (paraventricular, dorsomedial, and lateral hypothalamus), thalamus (paraventricular and centromedian thalamus), and limbic system (amygdala and nucleus accumbens). These regions influence numerous aspects of autonomic and neuroendocrine activity and are typically active during periods of wakefulness or arousal. By contrast, c-Fos was absent from the ventrolateral preoptic area (active during sleep). In standard behavioral tests (open field and elevated plus maze), mRGC activation induced behaviors commonly interpreted as anxiety like or as signs of increased alertness. Similar changes in behavior could be induced by bright light in wild-type and rodless and coneless mice, but not melanopsin knockout mice. These data demonstrate that mRGCs drive a light-dependent switch in behavioral motivation toward a more alert, risk-averse state. They also highlight the ability of this small fraction of retinal ganglion cells to realign activity in brain regions defining widespread aspects of physiology and behavior.

## Highlights

• Chemogenetics is used to recreate a single visual sensation in dark-housed animals
• mRGC activation alone resets circadian phase and constricts the pupil
• mRGC activation excites numerous thalamic, hypothalamic, and limbic brain regions
• mRGC activation realigns behavioral state toward alertness and/or anxiety

## Teaser

Milosavljevic et al. use chemogenetics to recreate the sensation of bright background light in mice held in darkness. This selective modulation resets the circadian clock; activates thalamic, hypothalamic, and limbic nuclei controlling diverse aspects of behavior and physiology; and induces a behavioral state of increased alertness and/or anxiety.

## Results and Discussion

**Figure 1:**
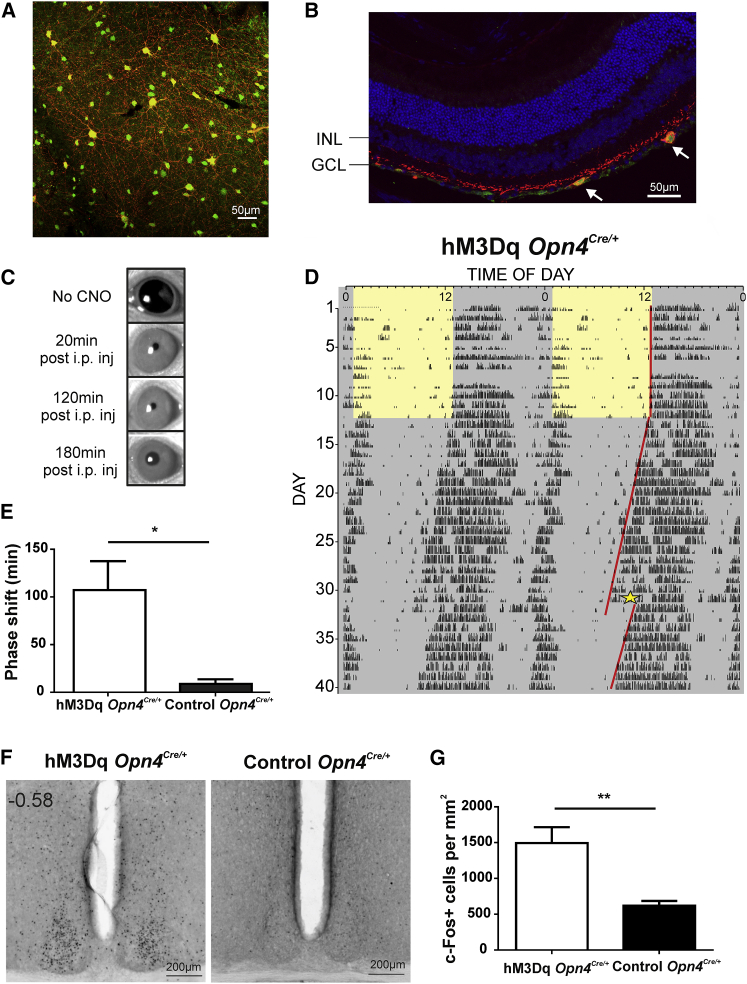
Chemogenetic Activation of mRGCs (A and B) After intravitreal injection of a viral vector (AAV2-hSyn-DIO-hM3Dq-mCherry) to Opn4^Cre^:Z/EGFP mice, immunohistochemical staining revealed transgene (mCherry; red) expression in GFP-positive neurons (green) in an en face view of retinal whole mounts (A) and retinal section (B). The retinal section shows the expression of transgene in cells of the retinal ganglion and inner nuclear cell layers (GCL and INL) of hM3Dq *Opn4*^*Cre/+*^ mice. Notice the different soma sizes of transduced cells (arrows), with DAPI stain in blue. A monochrome version of mCherry staining and more details on hM3Dq expression are provided in Figure S1. (C) Representative images of eyes under infrared illumination from hM3Dq-expressing mice held in darkness, prior to (top) and at 20, 120, and 180 min after intraperitoneal (i.p.) injection of CNO (5 mg/kg). For more details, see Figure S2. (D) Representative double-plotted actogram of wheel running activity of a hM3Dq mouse housed under a light:dark cycle (the light phase is indicated in yellow) until day 12 followed by constant darkness. The star shows the start of a 2 hr presentation of 0.25 mg/mL CNO in drinking water; the red line to left represents the eye fit through activity onsets. A representative double-plotted actogram of a control mouse is shown in Figure S3. (E) Change in circadian phase CNO application to hM3Dq-expressing (open bars; n = 4) and control (filled bars; n = 5) mice (means ± SEM; Mann-Whitney U test, p = 0.015). (F) Representative micrographs of coronal section through the SCN labeled for c-Fos (dark) from hM3Dq and control mice after CNO administration (5 mg/kg, i.p., at CT14). (G) Mean (±SEM) number of c-Fos positive cells mm^−2^ in SCN sections from hM3Dq (n = 6) and control (n = 6) mice (two-tailed unpaired t test,^∗∗^p < 0.01).

Determining whether melanopsin-expressing retinal ganglion cells (mRGCs) control behavioral state is complicated by the fact that their natural stimulus (bright background light) also allows numerous other visual responses to be engaged. One solution is to study mice lacking rods and cones, in which melanopsin (and therefore mRGCs) provides the only visual signal [10]. However, such preparations have substantial retinal reorganization [11], making them an imperfect representation of the intact system. Here, we therefore developed a method of acutely and selectively activating mRGCs in darkness in animals with a fully intact retina. Our approach was to target expression of the Gq-coupled chemogenetic tool hM3Dq (which reliably induces depolarization of neurons after administration of clozapine N-oxide [CNO] [12]) to mRGCs. Application of CNO should then mimic the excitatory effect of light for mRGCs without producing any other visual experience. To this end, we injected an AAV2 vector carrying a FLEX-switched hM3Dq coding sequence into the vitreous of *Opn4*^*Cre/+*^ mice. Selective expression of hM3Dq in mRGCs was confirmed in retinal whole mounts (Figures 1A and S1A) and sections (Figures 1B and S1B). Across nine retinas, 35% ± 7.5% (mean ± SD) of mRGCs expressed hM3Dq (mCherry marker), whereas all 722 hM3Dq-expressing cells detected were mRGCs (GFP marker). The distribution of soma size and projection pattern of transduced cells (Figure S1) was similar to that of the total mRGC population [8, 13], implying that there was no strong bias in transduction efficiency between subtypes of mRGC.

To confirm that the expressed receptor was able to simulate the effects of light exposure for mRGC driven responses, we asked whether CNO induced pupil constriction and circadian phase shifts (both established mRGC endpoints) in animals kept in darkness. Intraperitoneal injection of CNO (5 mg/kg) in mice unilaterally expressing hM3Dq induced strong bilateral pupil constriction within 20 min of application and persisting for at least 120 min (Figure 1C; ∼90% constriction, equivalent to the effect of 10^16^ photons/cm^2^/s; Figures S2A–S2D). To assess effects on the circadian clock, we recorded wheel-running behavior from animals free running in constant darkness and presented with 0.25 mg/mL CNO in sweetened drinking water (0.2% saccharine and 4% sucrose) for 2 hr at circadian time 14 (CT14). Mice drank between 650 and 800 μL (mean ± SD: 750 ± 58 μL and 700 ± 89 μL for hM3Dq and control, respectively), corresponding to 5–6.25 mg/kg of CNO. This treatment had the same effect as a bright light pulse at this time of night [14], delaying the circadian rhythm by 107 ± 30 min (Figures 1D and 1E). Neither of these effects of CNO administration constricted the pupil or shifted the clock in control mice lacking hM3Dq expression (Figures 1E, S2B, and S2E).

**Figure 2:**
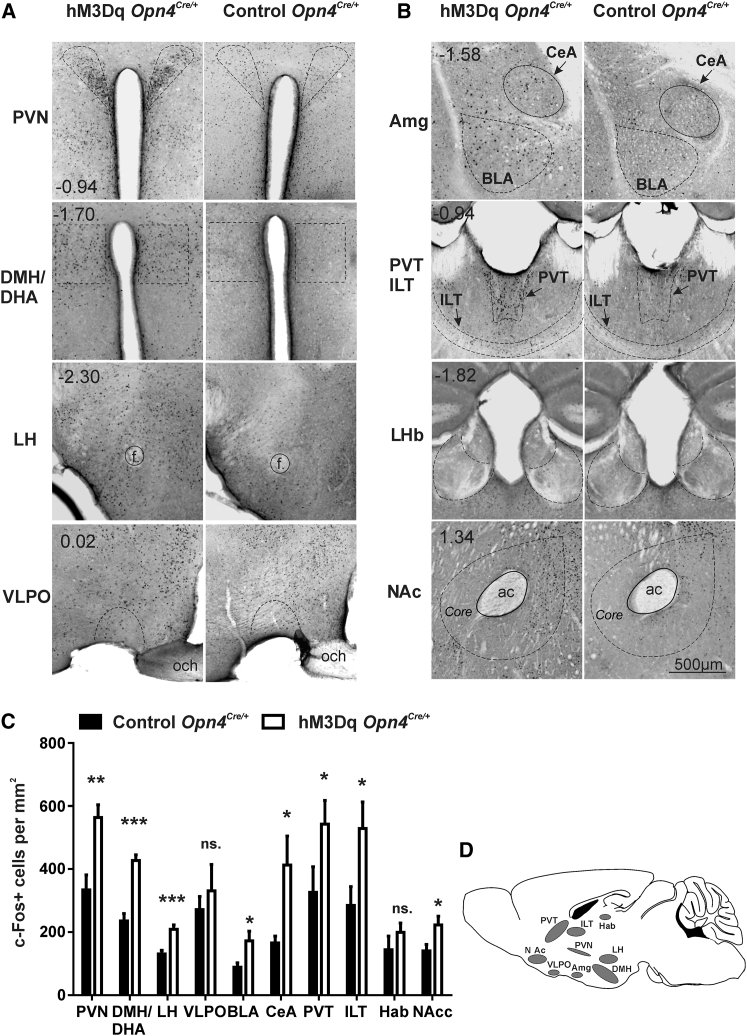
c-Fos Activity Mapping after Chemogenetic Activation of mRGCs (A and B) Representative micrographs of coronal sections showing c-Fos labeling (dark) in (A) the paraventrical hypothalamic nucleus (PVN), the dorsomedial hypothalamus/dorsal hypothalamic area (DMH/DHA), lateral (perifornical) hypothalamic area (LH), and ventrolateral preoptic nucleus (VLPO); and (B) amygdala (Amg), intralaminar thalamic nuclei (ITL), paraventricular thalamus (PVT), lateral habenula (LHb), and nucleus accumbens (NAc). Images to right of each panel are from control mice, and those to left are from unilateral hM3Dq-expressing animals (transduced eye to right of presented image, except in LH, VLPO, Amg, and NAc, where only the contralateral region is shown). (C) Mean (±SEM) number of c-Fos-positive cells mm^−2^ in all brain regions (bilaterally) after CNO administration (5 mg/kg, i.p., at CT14) in hM3Dq-expressing (open bars; n = 8) and control (filled bars; n = 8) mice (two-tailed unpaired t test, ^∗^p < 0.05, ^∗∗^p < 0.01, ^∗∗∗^p < 0.001). A complete summary of c-Fos data is provided in Table S1. BLA, basolateral amygdala; CeA, central nucleus of the amygdala. (D) Brain diagram illustrating target areas analyzed.

We next used induction of the immediate early gene, c-Fos, to identify brain regions excited by mRGC activation. In mice with unilateral hM3Dq expression, CNO injection (at CT14) induced strong bilateral c-Fos expression in the suprachiasmatic nuclei (SCN; the site of the master circadian clock and major hypothalamic target of mRGCs; Figures 1F and 1G). Elsewhere in the hypothalamus, we found significant bilateral c-Fos induction in the paraventricular hypothalamic nucleus (PVN), dorsomedial hypothalamus/dorsal hypothalamic area (DMH/DHA), and lateral hypothalamic area (LH) (Figures 2A and 2C). Together, these regions have access to very wide-ranging aspects of physiology and behavior. The PVN is one of the most important hypothalamic control centers, containing neuroendocrine neurons fundamental for hormonal regulation and homeostasis (hypothalmic-pituitary -adrenal [HPA] and -thyroid [HPT] axis, vasopressin, and oxytocin) and autonomic neurons connected to parasympathetic and sympathetic centers in brainstem and spinal cord. The DMH/DHA is also implicated in corticosteroid secretion and thermoregulation and, together with the LH, in controlling locomotor activity and feeding [15, 16, 17, 18, 19]. As a group (PVN, DMH/DHA, and LH), they are excited during periods of wakefulness [15, 20]. Accordingly, despite receiving a sparse mRGC projection, we found no significant c-Fos induction in the sleep activating ventrolateral preoptic area (VLPO) (Figure 2C) [8].

Turning to regions outside of the hypothalamus, we explored the amygdala, as this has been reported to be light activated in humans [7] and also to receive inputs from mRGCs [8]. We indeed found enhanced bilateral c-Fos in both basolateral (BLA) and central (CeA) amygdala (Figures 2B and 2C). Within the thalamus (and leaving aside conventional visual areas), c-Fos induction was restricted to the paraventricular thalamic (PVT) and intralaminar thalamic nuclei (ILT) (Figures 2B and 2C). Both the PVT and ILT have been independently associated with enhanced alertness and vigilance [21, 22], and the PVT plays a key role in energy homeostasis, arousal, temperature modulation, endocrine regulation, and reward [23]. The centromedian nucleus, a part of the ILT, receives a sparse input from mRGCs [9].

Previous work with chronically disrupted light exposure has revealed a depressive state in mice associated with activation of the lateral habenula [24]. We wondered whether acute activation of mRGCs had a similar effect. In fact, we did not find significant c-Fos induction in the lateral habenula (Figures 2B and 2C). Conversely, c-Fos was enhanced in the nucleus accumbens, a region typically associated with enhanced motivation and reward-seeking behavior [25].

**Figure 3:**
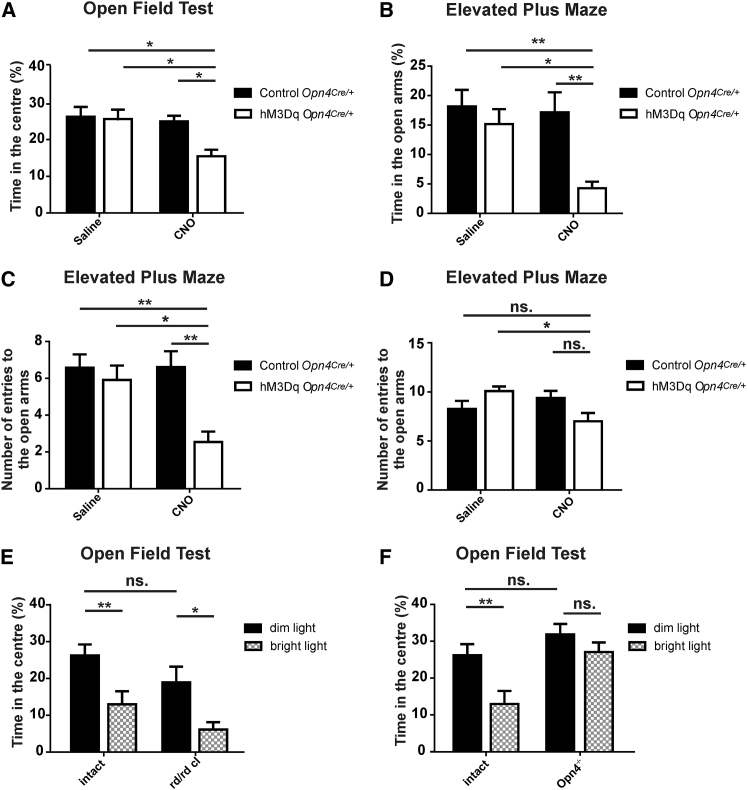
Chemogenetic and Light Activation of mRGCs Alters Performance in Behavioral Tests (A) Time spent in center over 10 min under dim far-red illumination in an open arena in hM3Dq-expressing and control *Opn4*^*Cre/+*^ mice treated with CNO or saline. Open bars depict data from hM3Dq-expressing mice, and filled bars depict data from control *Opn4*^*Cre/+*^ mice. (B–D) Time spent in open arms (B) and the number of entries to open (C) and closed (D) arms of an elevated plus maze under dim far-red illumination in hM3Dq and control *Opn4*^*Cre/+*^ mice treated with CNO or saline. (E) Time spent by visually intact (*Opn4*^*Cre/+*^) and rodless and coneless (*rd1;Cnga3*^*−/−*^) mice in center of an open arena under bright or dim far-red light. (F) Time spent by visually intact (*Opn4*^*Cre/+*^) and melanopsin knockout (*Opn4*^*−/−*^) mice in center of an open arena under bright or dim far-red light. All behavioral tests undertaken between CT14 and CT17; n = 9–13 per group, two-way ANOVA with post hoc Bonferroni correction, ^∗^p < 0.05, ^∗∗^p < 0.01. All graphs depict mean ± SEM, with open bars depicting hM3Dq-expressing mice and closed bars depicting control *Opn4*^*Cre/+*^ mice, except in (E) and (F), where closed bars depict dim light and hatched bars depict bright light. In all cases, dim far-red light was 2.91 μW cm^−2^, λ > 680 nm, and bright light was white, 217 μW cm^−2^.

In order to test whether these neurophysiological events translate to a change in behavioral state, we next tested the impact of selective mRGC activation on a battery of behavioral tests under dim far-red light (2.91 μW cm^−2^, λ > 680 nm). The open field test (OFT) is based on natural exploratory behavior of rodents in a novel open arena and widely used to measure mood changes in rodents. Anxiety-like behavioral states are associated with a reduced fraction of time spent in the center of the arena, an outcome that is independent of changes in total activity [26, 27, 28]. It has previously been reported that bright light induces such a response in rats [29]. We tested CNO-treated mice with bilateral expression of hM3Dq in mRGCs and found that they spent significantly less time in the center than controls (Figure 3A), although their overall activity was unaffected (total distance traveled, mean ± SEM: for control mice, 3,082 ± 245 cm and 3,369 ± 272 cm; for hM3Dq mice, 3,843 ± 260 cm and 3,384 ± 275 cm; saline and CNO, respectively; p > 0.05, two-way ANOVA).

For an independent assessment of mRGC-induced mood changes, we turned to an elevated plus maze (EPM), in which risk-averse states are reflected in avoidance of open arms. Again, hM3Dq-expressing mice treated with CNO entered open arms less frequently and spent less time in open arms than did controls (Figures 3B and 3C). There was a suggestion that they entered closed arms less frequently, indicating an overall reduction in activity, but this difference was not significant against all controls (Figure 3D).

Chronic disruption of light:dark cycles has been reported to induce a depressive state in mice [24]. However, CNO-treated hM3Dq-expressing mice showed no increased tendency toward helplessness (immobility) when subjected to a forced swim test (mean ± SEM, percentage time immobile: for control mice, 64.0% ± 10% and 56.3% ± 7.8%; for hM3Dq-expressing mice 46.5% ± 5.9% and 40.3% ± 6.1%; saline and CNO treatments, respectively; p > 0.05, two-way ANOVA). This argues that the immediate effects of mRGC activation do not extend to inducing a depressive state and that the published work in this area instead relates to the effects of chronic aberrant light exposure.

Collectively, these data reveal that selective activation of mRGCs has immediate effects on diverse brain structures and changes behavioral state. The range of brain regions showing c-*fos* induction after CNO administration in our hM3Dq-expressing mice is consistent with those implicated in changing mood and attention in humans based upon neuroimaging studies. The multiple functions of the sub-cortical structures involved allow the possibility that numerous behavioral and neuroendocrine systems may be engaged by this signal.

The overall effect of chemogenetic activation of mRGCs on behavioral state seems to be an increase in alertness and/or anxiety. The behavioral changes in OFT and EPM tests are considered indicators of enhanced risk aversion and commonly defined as anxiety like. However, as autonomic activation and increased arousal are among the earliest events observed in a state of anxiety, they could also be interpreted as increased attention or alertness [30]. Moreover, the chemogenetic manipulation induced c-Fos not in the sleep-active VLPO, but rather in the DMH/DHA (known to provide inhibitory control of the VLPO [16]), and in a range of other regions typically thought to be active during wakefulness (DMH/DHA, LH, and PVN) and arousal (amygdala, PVT, and ILT).

Thus, our c-*fos* and behavioral data both indicate an increase in anxiety and/or arousal after mRGC activation. However, previous studies have suggested that bright light has the opposite effect of inducing sleep [31, 32, 33]. To resolve this apparent contradiction, we finally tested the response of mice to bright light when challenged with the open field test. We found that in the light, wild-type (*Opn4*^*Cre/+*^, visually intact) mice showed the same reduction in time in the center previously observed for CNO-treated hM3Dq-expressing mice (Figures 3E and 3F; total distance traveled, mean ± SEM: 1,669 ± 178 cm and 3,082 ± 245 cm in the bright and dim far-red light, respectively). This anxiogenic effect of light was recapitulated in rodless and coneless mice (*rd*^*1*^*;Cnga3*^*−/−*^; Figure 3E; total distance traveled, mean ± SEM: 2,000 ± 225 cm and 3,229 ± 271 cm in the bright and dim far-red light, respectively) but was absent in mice lacking melanopsin (*Opn4*^*−/−*^; Figure 3F; total distance traveled, mean ± SEM: 3,286 ± 357 cm and 4,728 ± 585 cm in light and dark, respectively). The latter data are similar to those from a very recent study comparing responses to “blue” light in wild-type and melanopsin knockout mice [34].Together, they confirm that light can have arousal and/or anxiogenic effects and that mRGCs are both sufficient and necessary for this response. The ethological significance of such divergent light responses is unclear, but the observation that the appropriate response to a sensory stimulus may be context dependent is not in itself surprising. An arousal response would have an obvious survival advantage in mice for whom exposure to bright light would invariably indicate a situation of heightened danger. More importantly, it indicates that under the right circumstances, mRGCs can drive a change in mouse behavioral state analogous to the increase in alertness and arousal experienced by humans.

## Author Contributions

N.M. and R.J.L. designed the research. N.M. performed retinal and brain histology and behavioral experiments. N.M. and J.C.K. performed intravitreal injections and pupillometry. J.C.K. assisted with retinal histology. C.A.P. assisted with c-Fos immunohistochemistry and confocal microscopy. N.M. and R.J.L. wrote the manuscript with input from all authors.

## Supplemental Information

Document S1. Supplemental Experimental Procedures, Figures S1–S3, and Table S1

Document S2. Article plus Supplemental Information
