# Supplementary material for: Chemogenetic Activation of Melanopsin Retinal Ganglion Cells Induces Signatures of Arousal and/or Anxiety in Mice
Source: Curr Biol. 2016 Sep 12;26(17):2358–63. doi: 10.1016/j.cub.2016.06.057 (PMC5026697; doi:10.1016/j.cub.2016.06.057)
Supplement: Document S1. Supplemental Experimental Procedures, Figures S1–S3, and Table S1 [file mmc1.pdf]

Current Biology, Volume *26*

Supplemental Information

Chemogenetic Activation of Melanopsin Retinal Ganglion Cells Induces Signatures of Arousal and/or Anxiety in Mice

Nina Milosavljevic, Jasmina Cehajic-Kapetanovic, Christopher A. Procyk, and Robert J. Lucas

Figure S1 (Related to Figure 1)

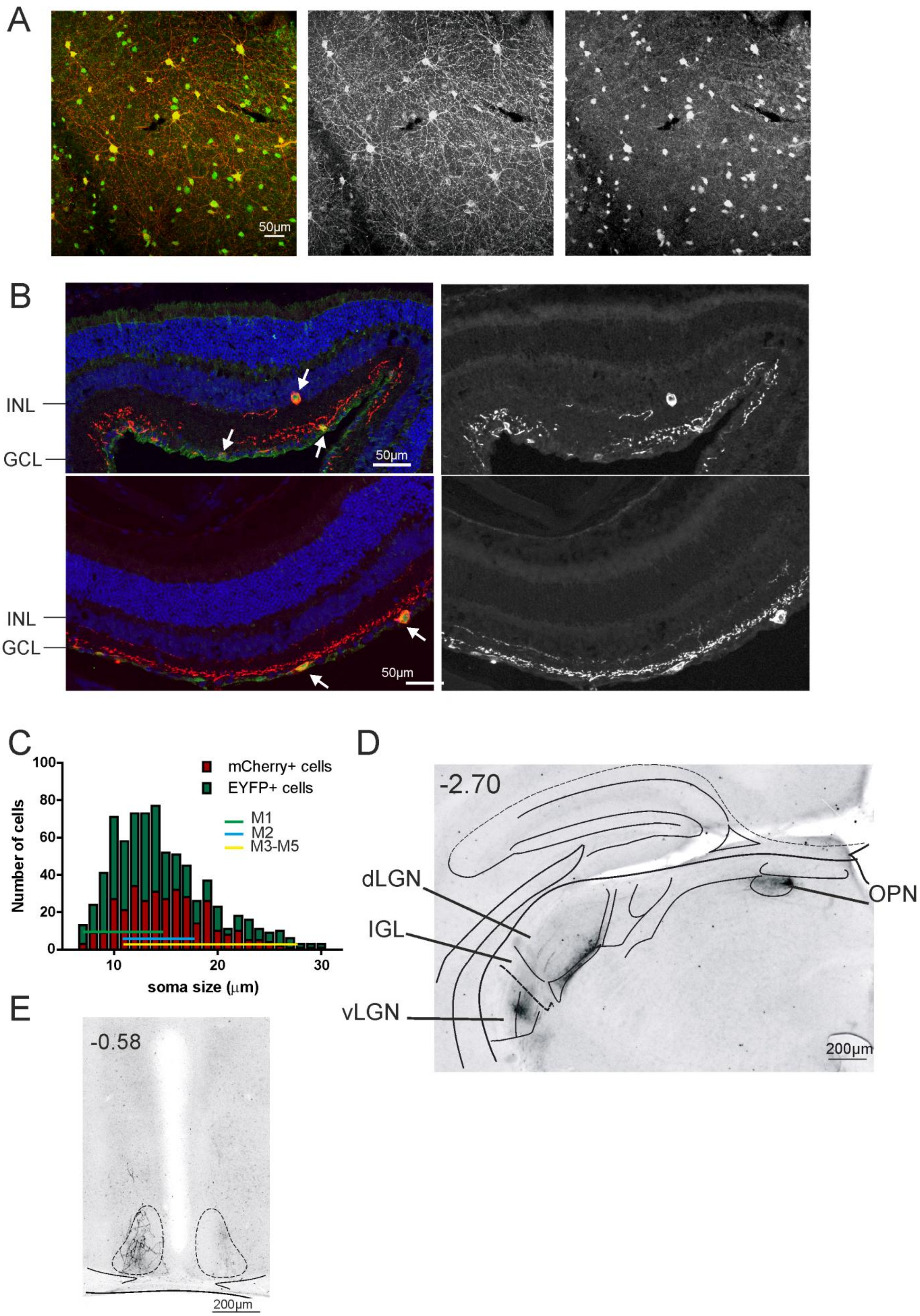

Figure S1. Related to Figure 1. Transgene expression following viral transduction of *Opn4*$^{Cre/+}$ retina. **A**) Examplar retinal wholemount from Figure 1A with monochrome versions of mCherry and GFP staining, from left to right. **B**) Examplar retinal sections from Figure 1 with monochrome versions of mCherry staining on the right. Notice the different soma sizes of transduced cells (white arrows) and a cell in INL, location of displaced M1 mRGCs [S2] and mCherry signal in OFF sublamina of IPL, DAPI stain in blue. **C**) Distribution of soma sizes of 335 mCherry+ cells and 754 EYFP+ cells sampled from 4 retinal wholemounts. Lines in different colors indicate expected range of soma sizes for different subtypes of mRGCs, green M1, blue M2 and yellow M3-M5[S1]. **D**) Coronal brain section of *Opn4*$^{Cre/+}$ mouse following injection of hM3Dq:mCherry virus in right eye shows mCherry signal (black) in axons of transduced retinal ganglion cells in oliviary pretectal nucleus (OPN), dorsal lateral geniculate nucleus (dLGN), intergeniculate leaflet (IGL) and ventral lateral geniculate nucleus (vLGN) at ~−2.70 mm from bregma. Approximate boundaries of nuclei in black from *Franklin KBJ and Paxinos G, the mouse brain, 2007*. **E**) Coronal brain section of *Opn4*$^{Cre/+}$ mouse following injection of hM3Dq-mCherry virus in right eye shows mCherry+ve processes (black) in SCN at ~−0.58 mm from bregma. The presence of mCherry processes in both SCN and OPN, and the ability of CNO to induce both pupil constriction and circadian phase shift indicates that both subtypes of M1 cells, Brn3b-positive and -negative M1 mRGCs, are trasduced[S1]. mCherry processes in other regions (along with soma size analysis) indicate M2-5 mRGC subtypes also transduced[S2].

Figure S2 (Related to Figure 1)

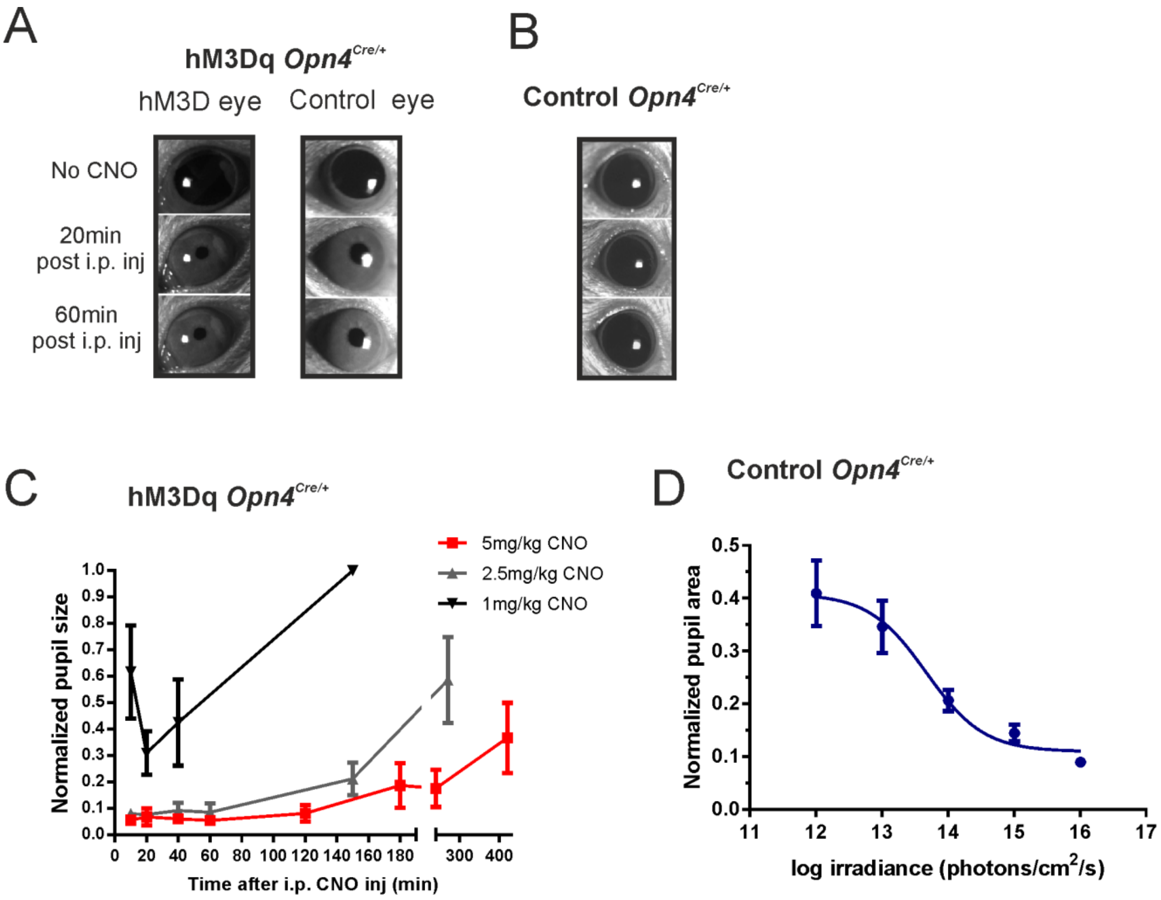

Figure S2. Related to Figure 1. Characterization of pupil response to chemogenetic activation of mRGCs. **A**) Representative frozen video images of eyes under infra-red illumination from an *Opn4*$^{Cre/+}$ mouse unilaterally expressing hM3Dq in mRGCs. Both hM3Dq and control eyes show strong constriction following CNO i.p. injection in complete darkness within first 20min that lasts for at least 120min. **B**) No change in pupil size was seen in a control *Opn4*$^{Cre/+}$ mouse treated with CNO. **C**) Mean(±SEM) pupil area (normalised to pre-CNO area=1) as a function of time after injection of CNO at 3 doses in hM3Dq-expressing *Opn4*$^{Cre/+}$ mice (n=6). **D**) Mean(±SEM) minimum normalised pupil area of control *Opn4*$^{Cre/+}$ under exposure to white light (10s) at different irradiances. Note that the pupil constriction induced by chemogenetic activation of mRGCs (C; ~90%) is equivalent to that produced by the brightest light (D), although given recent evidence that melanopsin can use a non Gq/11 phototransduction cascade [S3] the cellular mechanism may be different.

Figure S3 (Related to Figure 1)

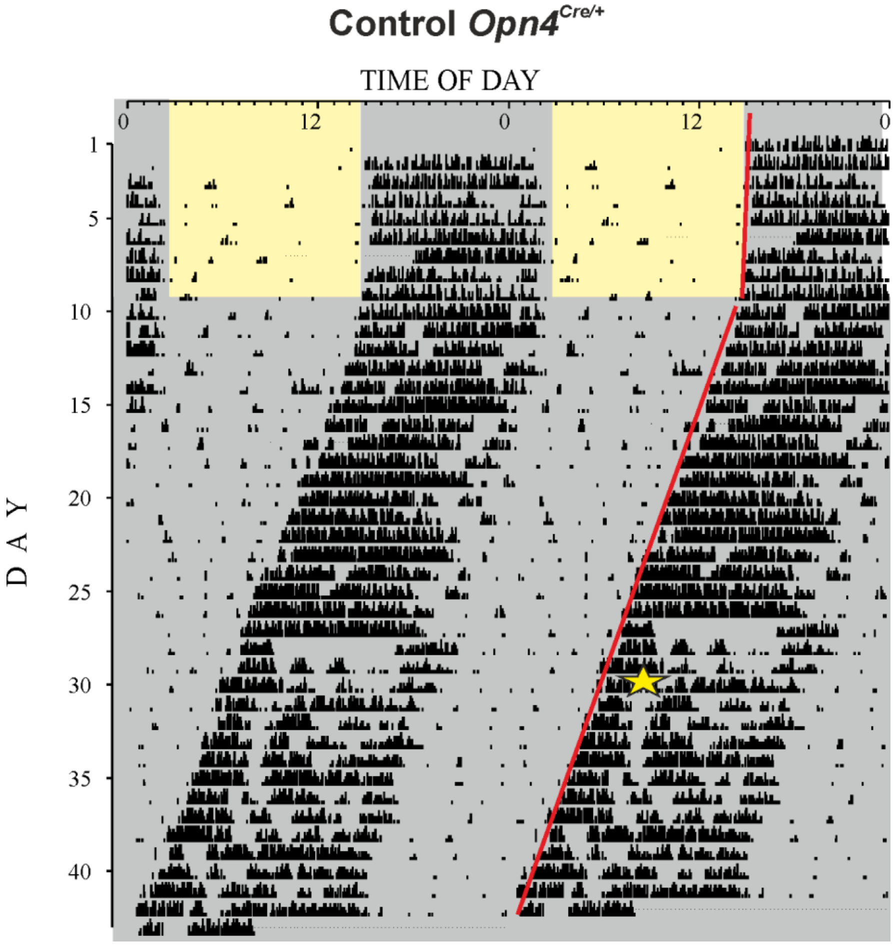

Figure S3. Related to Figure 1. Representative double-plotted actogram of wheel running activity of a control *Opn4*$^{Cre/+}$ mouse showing no change in activity to CNO administration at CT14 (asterisk designates time when a water bottle was replaced with the one containg CNO, 0.25mg/ml, for 2hrs).

Table S1. A complete statistical summary of c-Fos mapping data from Figure 2.

Brain region	hM3Dq *Opn4*$^{Cre/+}$	Control *Opn4*$^{Cre/+}$	Unpaired t-test p-values
Suprachiasmatic nuclei, SCN	1495.0 ± 220.8	618.5 ± 67.02	0.0020
Paraventricular hypothalamic nucleus, PVN	563.9 ± 39.92	334.0 ± 46.99	0.0023
Dorsomedial hypothalamus, DMH	427.4 ± 18.14	235.3 ± 23.18	0.0001
Lateral hypothalamic area, LH	209.0 ± 13.70	130.5 ± 11.42	0.0006
Ventrolateral preoptic nucleus, VLPO	331.3 ± 82.97	271.2 ± 41.77	0.5285
Basolateral amygdala, BLA	172.0 ± 31.10	88.84 ± 13.53	0.0279
Central nucleus of the amygdala, CeA	413.0 ± 91.67	165.8 ± 21.44	0.0200
Paraventricular nucleus of thalamus, PVT	521.9 ± 69.31	272.0 ± 78.93	0.0301
Intralaminar thalamic nuclei, ITL	528.8 ± 84.17	284.7 ± 59.28	0.0326
Nucleus accumbens, NAcc	229.9 ± 27.66	138.2 ± 20.76	0.0307
Lateral habenula, Hab	199.2 ± 29.73	144.3 ± 43.12	0.3193

Supplemental Experimental Procedures

Animals

Animal care was in accordance with the UK Animals, Scientific Procedures, Act (1986). Animals were kept in a 12-hour dark/light cycle at a temperature of 22ºC with food and water available *ad libitum*. Except where otherwise stated, experiments were performed on adult (4-8 months) *Opn4*$^{Cre/+}$ mice [S4]. Intravitreal injections of AAV2-hSyn-DIO-hM3Dq-mCherry vector (2.3x10^13 genomic particles/ml; The UNC Vector Core) were performed as previously reported [S5] and employed hyaluronan lyase and heparinase III (200U each) to maximise retinal penetration (total volume 2.5µl injected over 1 min). Control mice underwent the same procedure, with injections including glycosydic enzymes but with the virus replaced by vehicle (PBS). For c-Fos studies only the right eye was injected, while both eyes were injected in animals allocated to behavioural analyses. Mice were allowed at least 6 weeks to recover before being used in *in vivo* studies. Administration of Clozapine N-oxide (CNO; Abcam,) was performed via intraperitoneal (i.p.) route (5mg/kg), except where otherwise stated. The open field test was also undertaken on melanopsin knockouts (*Opn4*$^{-/-}$; [S6]) and rodless+coneless mice (*rd*^{1}*;Cnga3*$^{-/-}$) produced by crossing mice carrying the *rd*^{1} mutation of the pde6b gene (C57Bl6 strain), which lesions rod phototransduction and leads to photoreceptor degeneration, with Cnga3 knockouts that lack cone phototransduction [S7].

Immunohistochemistry

Immunohistochemistry was performed as previously described [S8] on retinal wholemounts and sections fixed in methanol-free 4% paraformaldehyde. The primary antibodies used in these studies include rabbit anti-dsRed (Clontech 632496; 1:1000) and chicken anti-GFP (Abcam ab13970; 1:1000). The secondary antibodies were Alexa 488 conjugated donkey anti-chicken (Jackson Immunoresearch) and Alexa 546 conjugated donkey anti-rabbit (Life Technologies) at 1:200. Images were collected on a Leica TCS SP5 AOBS inverted confocal microscope using a 40x/0.50 Plan Fluotar objective and 1.5x confocal zoom. Brain sections were imaged with Leica M165 FC Fluorescent Stereo Microscope.

c-Fos

Mice were housed under a 12h:12h LD cycle and both hM3Dq Opn4$^{Cre/+}$ and control Opn4$^{Cre/+}$ mice were i.p. injected with CNO (5mg/kg) at CT14 under dim, deep red light (50 nW cm^{-2}, λ>650nm), after which they were placed back in the dark for a further 90 min. Then, mice were deeply anaesthetized with i.p. injection of urethane (2.2g/kg; 30%w/v; Sigma-Aldrich, UK). Once anaesthetized, the mice were perfused transcardially with 0.9% saline followed by 4% paraformaldehyde. Brains were removed, postfixed overnight in 4% paraformaldehyde, and then transferred to 30% sucrose for cryoprotection for 2 days. Coronal brain sections (50µm) were stored free floating in 0.1 M phosphate buffer. Sections were incubated in 2% hydrogen peroxidase for 20min and then in blocking buffer (0.1 M phosphate buffer, 3% Triton X-100 and 0.5% normal goat serum) for 1 h. Sections were incubated in rabbit anti-c-Fos (Calbiochem Ab-5; 1:20,000) overnight at 4°C and then visualized with a goat anti-rabbit Vectastain horseradish peroxidase kit (Vector Labs) and DAB (Vector Labs) as a chromogen. Sections were mounted on microscope slides and coverslipped with DPX. Images were acquired using the 3D Histech Pannoramic 250 Flash II slide scanner with 20x/0.80 Plan Apo objective. 3D Histech Pannoramic Viewer and ImageJ were used to count c-Fos-positive cells and measure the area of region counted from, by a blinded scorer. Between 3-5 sections per nuclei are analysed matching the same coordinates, same part of nuclei, in different animals according to Franklin KBJ and Paxinos G, the mouse brain, 2007. The number of c-Fos positive cells was normalized to the area of the region. These data were analysed using a two-tailed unpaired t-test (GraphPad Prism 6.04).

Pupillometry

Pupils were filmed in dark under infrared illumination (IR LED >900nm) by a video camera (QImaging Rolera-XR) in unanaesthetised and gently restrained mice before and after CNO injection (5 mg/kg, i.p, except where otherwise stated). Videos were analysed using ImageJ software. The areas of the pupil after CNO administration were expressed relative to the area of the pupil before CNO injection. The PLR irradiance response curve was constructed for consensual response in control *Opn4*$^{Cre/+}$ mice using a 10s full field white (150 W Metal-halide lamp; Phillips) pulse intensity controlled using neutral density filters. White light was measured using a spectroradiometer (SpectroCAL classic spectroradiometer, Cambridge Research Systems), which measured the power in milliwatt per square centimetre at wavelengths between 350 and 750 nm. Then the total photons were calculated.

Wheel-running behaviour

To assess wheel-running behaviour in LD and constant darkness, mice were individually housed in cages equipped with a running wheel (82 mm diameter, Vet-Tech, UK) under a 12h:12h LD cycle for a minimum of

10 days then released into DD for at least 14 days. The intensity of light during lights-on was 239.45 µW cm^{-2}. For phase-shifting experiments, a standard water bottle was replaced with one containing 0.25mg/ml CNO with 0.2% saccharine and 4% sucrose at CT14 for 2h. During this time, mice consumed between 650-800µl (mean±STD, 750±58µl and 700±89µl for hM3Dq and control *Opn4*$^{Cre/+}$ mice respectively), corresponding to a dose range of 5-6.25 mg/kg. Wheel-running data were monitored throughout the experiment and recorded in 5 min time bins using the Chronobiology Kit (Stanford Software Systems, Santa Cruz, CA, USA). These results were compared between hM3Dq and control *Opn4*$^{Cre/+}$ mice treated with CNO using Mann-Whitney U test, (GraphPad Prism 6.04).

Behaviour
hM3Dq *Opn4*$^{Cre/+}$ mice had bilateral intravitreal injections of AAV2-hM3Dq and control *Opn4*$^{Cre/+}$ mice had mock intravitreal injections with PBS instead of virus. Recordings were undertaken in their subjective night (between CT14 and CT17). Except where otherwise stated experiments were conducted under far red light (2.91 µW cm^{-2}, λ>680nm). Mice were injected i.p. with CNO (5mg/kg) or saline 30min before recording started. In light vs dark experiments, mice were exposed to light for 30min before the open field test experiments.

Open field
Mice were individually placed in the centre of a square arena (40 × 40 x 30cm) and allowed to explore for 10 min. Central zone is 40% of the total surface (8cm from the edge of the arena walls). Behaviour was monitored from above by a video camera. The apparatus was cleaned thoroughly between each trial. Distance travelled and time in the centre of the arena were measured using video tracking system (EthoVision XT10). These measures were compared between hM3Dq *Opn4*$^{Cre/+}$ mice injected with CNO with all the controls by two-way ANOVA with post hoc Bonferroni correction (GraphPad Prism 6.04). Experiments in Opn4$^{Cre/+}$, rodless+coneless (rd^{1}, Cnga3$^{-/-}$) and melanopsin knockout mice (Opn4$^{-/-}$) were performed under far red light (2.91 µW cm^{-2}, λ>680nm) and bright light (217 µW cm-2, halogen source).

Elevated plus maze
The apparatus consisted of two open arms (25 x 5cm) and two arms enclosed by walls (25 x 5 x 16cm) forming a cross. The arms were separated by a central platform (5 × 5 cm). The maze was elevated (52 cm). Mice were placed in the centre of the elevated plus maze facing one of the open arms that was kept consistent between mice. Behaviour was monitored from above by a video camera. The apparatus was cleaned thoroughly between each trial. Video tracking system (EthoVision XT10) was used. The time spent and number of entries in the open arms were measured as indications of risk averse behaviour. These results were compared between hM3Dq *Opn4*$^{Cre/+}$ mice treated with CNO and all the controls using two-way ANOVA followed by a Bonferroni post-hoc test (GraphPad Prism 6.04).

Forced swim test
Mice were individually placed in an inescapable container (35cm height, 20cm diameter) of water (25°C) filled (up to 15cm) for 6 min. Behaviour was monitored by video cameras positioned in front of the apparatus and post recording manually scored. Time spent immobile for the last 4 min of the test was calculated. Increased time spent immobile is indicative of increased depression-like behaviour. The amount of time spent immobile during the last 4 min was analysed by two-way ANOVA with post hoc Bonferroni correction (GraphPad Prism 6.04).

Supplemental References:

S1. Chen, S.K., Badea, T.C., and Hattar, S. (2011). Photoentrainment and pupillary light reflex are mediated by distinct populations of ipRGCs. Nature *476*, 92-95.

S2. Berson, D.M., Castrucci, A.M., and Provencio, I. (2010). Morphology and mosaics of melanopsin-expressing retinal ganglion cell types in mice. J Comp Neurol *518*, 2405-2422.

S3. Chew, K.S., Schmidt, T.M., Rupp, A.C., Kofuji, P., and Trimarchi, J.M. (2014). Loss of gq/11 genes does not abolish melanopsin phototransduction. PLoS One *9*, e98356.

S4. Ecker, J.L., Dumitrescu, O.N., Wong, K.Y., Alam, N.M., Chen, S.K., LeGates, T., Renna, J.M., Prusky, G.T., Berson, D.M., and Hattar, S. (2010). Melanopsin-expressing retinal ganglion-cell photoreceptors: cellular diversity and role in pattern vision. Neuron *67*, 49-60.

S5. Cehajic-Kapetanovic, J., Le Goff, M.M., Allen, A., Lucas, R.J., and Bishop, P.N. (2011). Glycosidic enzymes enhance retinal transduction following intravitreal delivery of AAV2. Mol Vis *17*, 1771-1783.

S6. Lucas, R.J., Hattar, S., Takao, M., Berson, D.M., Foster, R.G., and Yau, K.W. (2003). Diminished pupillary light reflex at high irradiances in melanopsin-knockout mice. Science *299*, 245-247.

S7. Hattar, S., Lucas, R.J., Mrosovsky, N., Thompson, S., Douglas, R.H., Hankins, M.W., Lem, J., Biel, M., Hofmann, F., Foster, R.G., et al. (2003). Melanopsin and rod-cone photoreceptive systems account for all major accessory visual functions in mice. Nature *424*, 76-81.

S8. Storchi, R., Milosavljevic, N., Eleftheriou, C.G., Martial, F.P., Orlowska-Feuer, P., Bedford, R.A., Brown, T.M., Montemurro, M.A., Petersen, R.S., and Lucas, R.J. (2015). Melanopsin-driven increases in maintained activity enhance thalamic visual response reliability across a simulated dawn. Proc Natl Acad Sci U S A.
