# Supplementary material for: Chemogenetic Activation of Melanopsin Retinal Ganglion Cells Induces Signatures of Arousal and/or Anxiety in Mice
Source: Curr Biol. 2016 Sep 12;26(17):2358–63. doi: 10.1016/j.cub.2016.06.057 (PMC5026697; doi:10.1016/j.cub.2016.06.057)
Supplement: Document S2. Article plus Supplemental Information [file mmc2.pdf]

Report

Current Biology

Chemogenetic Activation of Melanopsin Retinal Ganglion Cells Induces Signatures of Arousal and/or Anxiety in Mice

Highlights

- Chemogenetics is used to recreate a single visual sensation in dark-housed animals
- mRGC activation alone resets circadian phase and constricts the pupil
- mRGC activation excites numerous thalamic, hypothalamic, and limbic brain regions
- mRGC activation realigns behavioral state toward alertness and/or anxiety

Authors

Nina Milosavljevic,
Jasmina Cehajic-Kapetanovic,
Christopher A. Procyk, Robert J. Lucas

Correspondence

nina.milosavljevic@manchester.ac.uk

In Brief

Milosavljevic et al. use chemogenetics to recreate the sensation of bright background light in mice held in darkness. This selective modulation resets the circadian clock; activates thalamic, hypothalamic, and limbic nuclei controlling diverse aspects of behavior and physiology; and induces a behavioral state of increased alertness and/or anxiety.

Milosavljevic et al., 2016, Current Biology *26*, 2358–2363
September 12, 2016 © 2016 The Authors. Published by Elsevier Ltd.
http://dx.doi.org/10.1016/j.cub.2016.06.057

Current Biology
Report

Chemogenetic Activation of Melanopsin Retinal Ganglion Cells Induces Signatures of Arousal and/or Anxiety in Mice

Nina Milosavljevic,[1,*] Jasmina Cehajic-Kapetanovic,[2] Christopher A. Procyk,[1] and Robert J. Lucas[1]
[1]Faculty of Life Sciences, University of Manchester, Oxford Road, Manchester M13 9PT, UK
[2]Centre for Ophthalmology and Vision Sciences, Institute of Human Development, University of Manchester, Manchester M13 9PT, UK
*Correspondence: nina.milosavljevic@manchester.ac.uk
http://dx.doi.org/10.1016/j.cub.2016.06.057

SUMMARY

Functional imaging and psychometric assessments indicate that bright light can enhance mood, attention, and cognitive performance in humans. Indirect evidence links these events to light detection by intrinsically photosensitive melanopsin-expressing retinal ganglion cells (mRGCs) [1–9]. However, there is currently no direct demonstration that mRGCs can have such an immediate effect on mood or behavioral state in any species. We addressed this deficit by using chemogenetics to selectively activate mRGCs, simulating the excitatory effects of bright light on this cell type in dark-housed mice. This specific manipulation evoked circadian phase resetting and pupil constriction (known consequences of mRGC activation). It also induced c-Fos (a marker of neuronal activation) in multiple nuclei in the hypothalamus (paraventricular, dorsomedial, and lateral hypothalamus), thalamus (paraventricular and centromedian thalamus), and limbic system (amygdala and nucleus accumbens). These regions influence numerous aspects of autonomic and neuroendocrine activity and are typically active during periods of wakefulness or arousal. By contrast, c-Fos was absent from the ventrolateral preoptic area (active during sleep). In standard behavioral tests (open field and elevated plus maze), mRGC activation induced behaviors commonly interpreted as anxiety like or as signs of increased alertness. Similar changes in behavior could be induced by bright light in wild-type and rodless and coneless mice, but not melanopsin knockout mice. These data demonstrate that mRGCs drive a light-dependent switch in behavioral motivation toward a more alert, risk-averse state. They also highlight the ability of this small fraction of retinal ganglion cells to realign activity in brain regions defining widespread aspects of physiology and behavior.

RESULTS AND DISCUSSION

Determining whether melanopsin-expressing retinal ganglion cells (mRGCs) control behavioral state is complicated by the fact that their natural stimulus (bright background light) also allows numerous other visual responses to be engaged. One solution is to study mice lacking rods and cones, in which melanopsin (and therefore mRGCs) provides the only visual signal [10]. However, such preparations have substantial retinal reorganization [11], making them an imperfect representation of the intact system. Here, we therefore developed a method of acutely and selectively activating mRGCs in darkness in animals with a fully intact retina. Our approach was to target expression of the Gq-coupled chemogenetic tool hM3Dq (which reliably induces depolarization of neurons after administration of clozapine N-oxide [CNO] [12]) to mRGCs. Application of CNO should then mimic the excitatory effect of light for mRGCs without producing any other visual experience. To this end, we injected an AAV2 vector carrying a FLEX-switched hM3Dq coding sequence into the vitreous of *Opn4*$^{Cre/+}$ mice. Selective expression of hM3Dq in mRGCs was confirmed in retinal whole mounts (Figures 1A and S1A) and sections (Figures 1B and S1B). Across nine retinas, 35% ± 7.5% (mean ± SD) of mRGCs expressed hM3Dq (mCherry marker), whereas all 722 hM3Dq-expressing cells detected were mRGCs (GFP marker). The distribution of soma size and projection pattern of transduced cells (Figure S1) was similar to that of the total mRGC population [8, 13], implying that there was no strong bias in transduction efficiency between subtypes of mRGC.

To confirm that the expressed receptor was able to simulate the effects of light exposure for mRGC driven responses, we asked whether CNO induced pupil constriction and circadian phase shifts (both established mRGC endpoints) in animals kept in darkness. Intraperitoneal injection of CNO (5 mg/kg) in mice unilaterally expressing hM3Dq induced strong bilateral pupil constriction within 20 min of application and persisting for at least 120 min (Figure 1C; ~90% constriction, equivalent to the effect of 10^{16} photons/cm^2/s; Figures S2A–S2D). To assess effects on the circadian clock, we recorded wheel-running behavior from animals free running in constant darkness and presented with 0.25 mg/mL CNO in sweetened drinking water (0.2% saccharine and 4% sucrose) for 2 hr at circadian time 14 (CT14). Mice drank between 650 and 800 µL (mean ± SD: 750 ± 58 µL and 700 ± 89 µL for hM3Dq and control,

 © 2016 The Authors. Published by Elsevier Ltd.
This is an open access article under the CC BY license (http://creativecommons.org/licenses/by/4.0/).

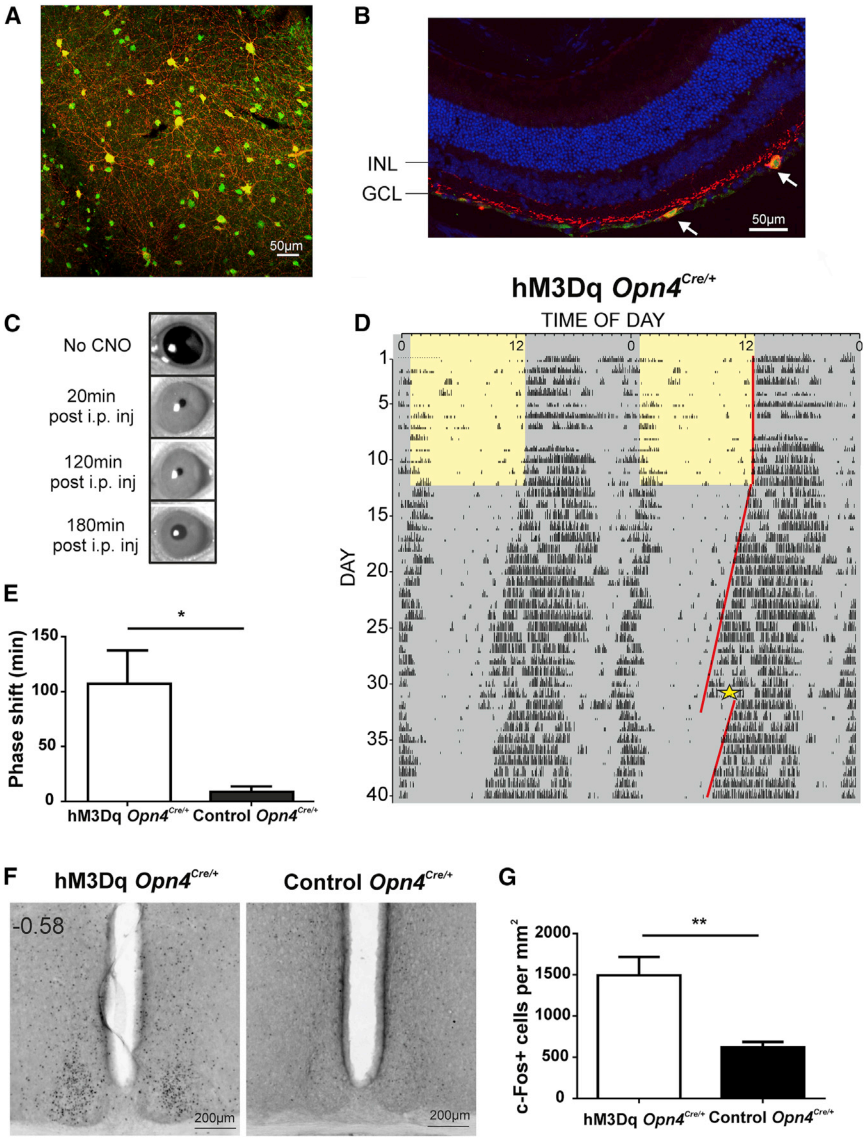

Figure 1. Chemogenetic Activation of mRGCs

(A and B) After intravitreal injection of a viral vector (AAV2-hSyn-DIO-hM3Dq-mCherry) to $Opn4^{Cre}$:Z/EGFP mice, immunohistochemical staining revealed transgene (mCherry; red) expression in GFP-positive neurons (green) in an en face view of retinal whole mounts (A) and retinal section (B). The retinal section shows the expression of transgene in cells of the retinal ganglion and inner nuclear cell layers (GCL and INL) of hM3Dq $Opn4^{Cre/+}$ mice. Notice the different soma sizes of transduced cells (arrows), with DAPI stain in blue. A monochrome version of mCherry staining and more details on hM3Dq expression are provided in Figure S1.

(C) Representative images of eyes under infrared illumination from hM3Dq-expressing mice held in darkness, prior to (top) and at 20, 120, and 180 min after intraperitoneal (i.p.) injection of CNO (5 mg/kg). For more details, see Figure S2.

(D) Representative double-plotted actogram of wheel running activity of a hM3Dq mouse housed under a light:dark cycle (the light phase is indicated in yellow) until day 12 followed by constant darkness. The star shows the start of a 2 hr presentation of 0.25 mg/mL CNO in drinking water; the red line to left represents the eye fit through activity onsets. A representative double-plotted actogram of a control mouse is shown in Figure S3.

(E) Change in circadian phase CNO application to hM3Dq-expressing (open bars; n = 4) and control (filled bars; n = 5) mice (means ± SEM; Mann-Whitney U test, p = 0.015).

(F) Representative micrographs of coronal section through the SCN labeled for c-Fos (dark) from hM3Dq and control mice after CNO administration (5 mg/kg, i.p., at CT14).

(G) Mean (±SEM) number of c-Fos positive cells mm^{-2} in SCN sections from hM3Dq (n = 6) and control (n = 6) mice (two-tailed unpaired t test,**p < 0.01).

respectively), corresponding to 5–6.25 mg/kg of CNO. This treatment had the same effect as a bright light pulse at this time of night [14], delaying the circadian rhythm by 107 ± 30 min (Figures 1D and 1E). Neither of these effects of CNO administration constricted the pupil or shifted the clock in control mice lacking hM3Dq expression (Figures 1E, S2B, and S2E).

We next used induction of the immediate early gene, c-Fos, to identify brain regions excited by mRGC activation. In mice with unilateral hM3Dq expression, CNO injection (at CT14) induced strong bilateral c-Fos expression in the suprachiasmatic nuclei (SCN; the site of the master circadian clock and major hypothalamic target of mRGCs; Figures 1F and 1G). Elsewhere in the hypothalamus, we found significant bilateral c-Fos induction in the paraventricular hypothalamic nucleus (PVN), dorsomedial hypothalamus/dorsal hypothalamic area (DMH/DHA), and lateral hypothalamic area (LH) (Figures 2A and 2C). Together, these regions have access to very wide-ranging aspects of physiology and behavior. The PVN is one of the most important hypothalamic control centers, containing neuroendocrine neurons fundamental for hormonal regulation and homeostasis (hypothalamic-pituitary -adrenal [HPA] and -thyroid [HPT] axis, vasopressin, and oxytocin) and autonomic neurons connected to parasympathetic and sympathetic centers in brainstem and spinal cord. The DMH/DHA is also implicated in corticosteroid secretion and thermoregulation and, together with the LH, in controlling locomotor activity and feeding [15–19]. As a group (PVN, DMH/DHA, and LH), they are excited during periods of wakefulness [15, 20]. Accordingly, despite receiving a sparse mRGC projection, we found no significant c-Fos induction in the sleep activating ventrolateral preoptic area (VLPO) (Figure 2C) [8].

Turning to regions outside of the hypothalamus, we explored the amygdala, as this has been reported to be light activated in humans [7] and also to receive inputs from mRGCs [8]. We indeed found enhanced bilateral c-Fos in both basolateral (BLA) and central (CeA) amygdala (Figures 2B and 2C). Within the thalamus

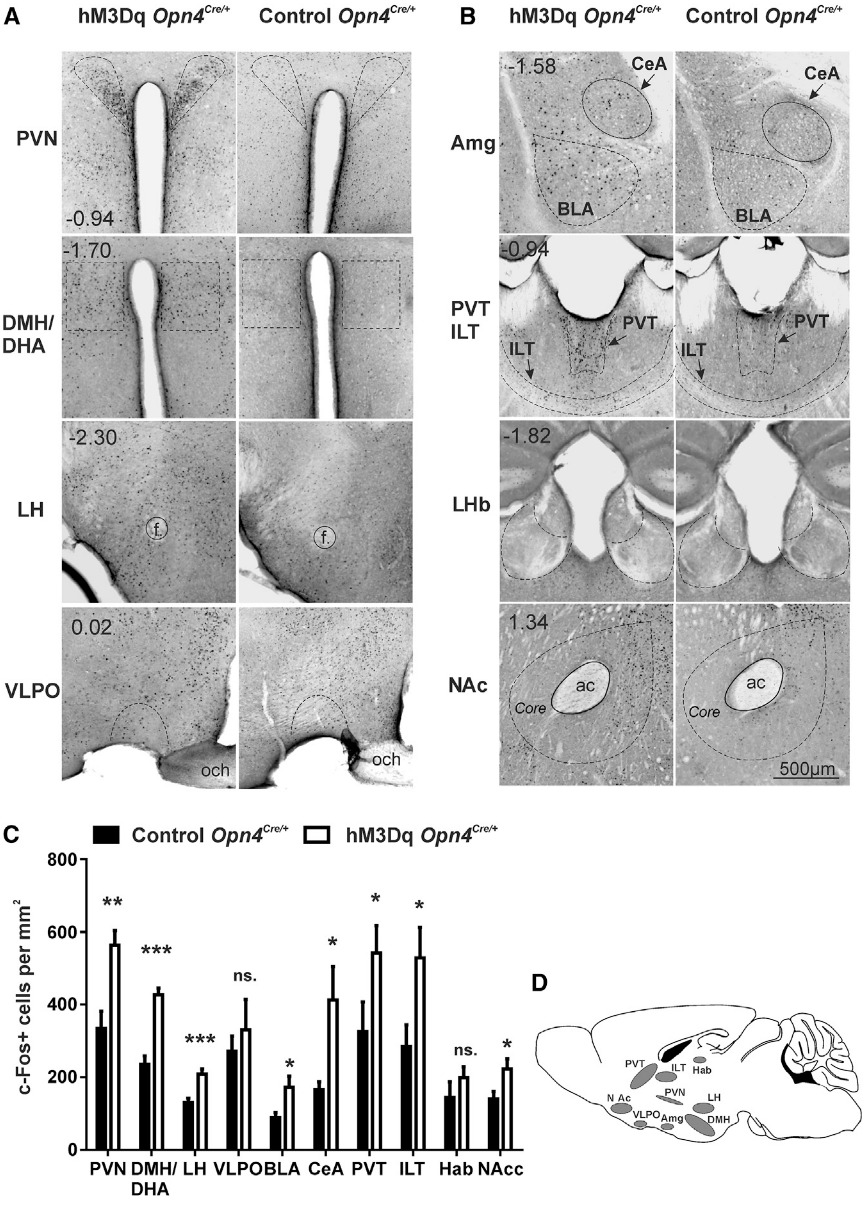

Figure 2. c-Fos Activity Mapping after Chemogenetic Activation of mRGCs

(A and B) Representative micrographs of coronal sections showing c-Fos labeling (dark) in (A) the paraventricular hypothalamic nucleus (PVN), the dorsomedial hypothalamus/dorsal hypothalamic area (DMH/DHA), lateral (perifornical) hypothalamic area (LH), and ventrolateral preoptic nucleus (VLPO); and (B) amygdala (Amg), intralaminar thalamic nuclei (ITL), paraventricular thalamus (PVT), lateral habenula (LHb), and nucleus accumbens (NAc). Images to right of each panel are from control mice, and those to left are from unilateral hM3Dq-expressing animals (transduced eye to right of presented image, except in LH, VLPO, Amg, and NAc, where only the contralateral region is shown).

(C) Mean (±SEM) number of c-Fos-positive cells mm^{-2} in all brain regions (bilaterally) after CNO administration (5 mg/kg, i.p., at CT14) in hM3Dq-expressing (open bars; n = 8) and control (filled bars; n = 8) mice (two-tailed unpaired t test, *$p < 0.05$, **$p < 0.01$, ***$p < 0.001$). A complete summary of c-Fos data is provided in Table S1. BLA, basolateral amygdala; CeA, central nucleus of the amygdala.

(D) Brain diagram illustrating target areas analyzed.

(and leaving aside conventional visual areas), c-Fos induction was restricted to the paraventricular thalamic (PVT) and intralaminar thalamic nuclei (ILT) (Figures 2B and 2C). Both the PVT and ILT have been independently associated with enhanced alertness and vigilance [21, 22], and the PVT plays a key role in energy homeostasis, arousal, temperature modulation, endocrine regulation, and reward [23]. The centromedian nucleus, a part of the ILT, receives a sparse input from mRGCs [9].

Previous work with chronically disrupted light exposure has revealed a depressive state in mice associated with activation of the lateral habenula [24]. We wondered whether acute activation of mRGCs had a similar effect. In fact, we did not find significant c-Fos induction in the lateral habenula (Figures 2B and 2C). Conversely, c-Fos was enhanced in the nucleus accumbens, a region typically associated with enhanced motivation and reward-seeking behavior [25].

In order to test whether these neurophysiological events translate to a change in behavioral state, we next tested the impact of selective mRGC activation on a battery of behavioral tests under dim far-red light (2.91 $\mu W\ cm^{-2}$, $\lambda > 680$ nm). The open field test (OFT) is based on natural exploratory behavior of rodents in a novel open arena and widely used to measure mood changes in rodents. Anxiety-like behavioral states are associated with a reduced fraction of time spent in the center of the arena, an outcome that is independent of changes in total activity [26–28]. It has previously been reported that bright light induces such a response in rats [29]. We tested CNO-treated mice with bilateral expression of hM3Dq in mRGCs and found that they spent significantly less time in the center than controls (Figure 3A), although their overall activity was unaffected (total distance traveled, mean ± SEM: for control mice, 3,082 ± 245 cm and 3,369 ± 272 cm; for hM3Dq mice, 3,843 ± 260 cm and 3,384 ± 275 cm; saline and CNO, respectively; $p > 0.05$, two-way ANOVA).

For an independent assessment of mRGC-induced mood changes, we turned to an elevated plus maze (EPM), in which risk-averse states are reflected in avoidance of open arms. Again, hM3Dq-expressing mice treated with CNO entered open arms less frequently and spent less time in open arms than did controls (Figures 3B and 3C). There was a suggestion that they entered closed arms less frequently, indicating an overall reduction in activity, but this difference was not significant against all controls (Figure 3D).

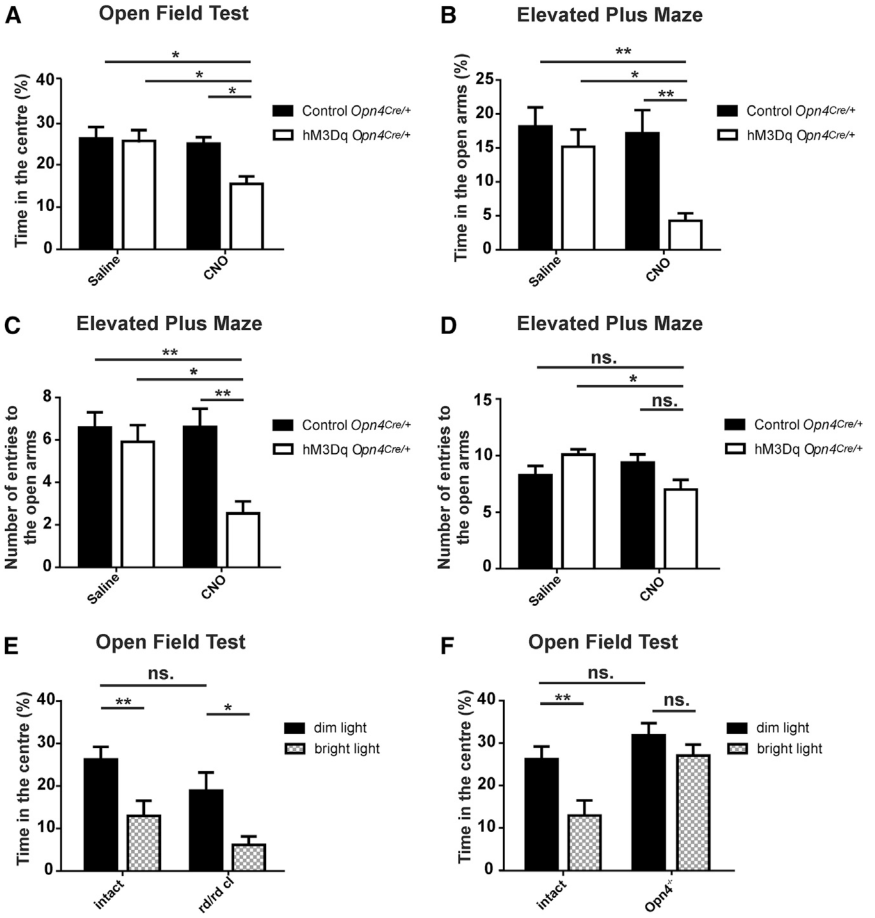

Figure 3. Chemogenetic and Light Activation of mRGCs Alters Performance in Behavioral Tests

(A) Time spent in center over 10 min under dim far-red illumination in an open arena in hM3Dq-expressing and control *Opn4*$^{Cre/+}$ mice treated with CNO or saline. Open bars depict data from hM3Dq-expressing mice, and filled bars depict data from control *Opn4*$^{Cre/+}$ mice.

(B–D) Time spent in open arms (B) and the number of entries to open (C) and closed (D) arms of an elevated plus maze under dim far-red illumination in hM3Dq and control *Opn4*$^{Cre/+}$ mice treated with CNO or saline.

(E) Time spent by visually intact (*Opn4*$^{Cre/+}$) and rodless and coneless (*rd1;Cnga3*$^{-/-}$) mice in center of an open arena under bright or dim far-red light.

(F) Time spent by visually intact (*Opn4*$^{Cre/+}$) and melanopsin knockout (*Opn4*$^{-/-}$) mice in center of an open arena under bright or dim far-red light.

All behavioral tests undertaken between CT14 and CT17; n = 9–13 per group, two-way ANOVA with post hoc Bonferroni correction, *$p < 0.05$, **$p < 0.01$. All graphs depict mean ± SEM, with open bars depicting hM3Dq-expressing mice and closed bars depicting control *Opn4*$^{Cre/+}$ mice, except in (E) and (F), where closed bars depict dim light and hatched bars depict bright light. In all cases, dim far-red light was 2.91 μW cm^{-2}, $\lambda > 680$ nm, and bright light was white, 217 μW cm^{-2}.

Chronic disruption of light:dark cycles has been reported to induce a depressive state in mice [24]. However, CNO-treated hM3Dq-expressing mice showed no increased tendency toward helplessness (immobility) when subjected to a forced swim test (mean ± SEM, percentage time immobile: for control mice, 64.0% ± 10% and 56.3% ± 7.8%; for hM3Dq-expressing mice 46.5% ± 5.9% and 40.3% ± 6.1%; saline and CNO treatments, respectively; $p > 0.05$, two-way ANOVA). This argues that the immediate effects of mRGC activation do not extend to inducing a depressive state and that the published work in this area instead relates to the effects of chronic aberrant light exposure.

Collectively, these data reveal that selective activation of mRGCs has immediate effects on diverse brain structures and changes behavioral state. The range of brain regions showing c-*fos* induction after CNO administration in our hM3Dq-expressing mice is consistent with those implicated in changing mood and attention in humans based upon neuroimaging studies. The multiple functions of the sub-cortical structures involved allow the possibility that numerous behavioral and neuroendocrine systems may be engaged by this signal.

The overall effect of chemogenetic activation of mRGCs on behavioral state seems to be an increase in alertness and/or anxiety. The behavioral changes in OFT and EPM tests are considered indicators of enhanced risk aversion and commonly defined as anxiety like. However, as autonomic activation and increased arousal are among the earliest events observed in a state of anxiety, they could also be interpreted as increased attention or alertness [30]. Moreover, the chemogenetic manipulation induced c-Fos not in the sleep-active VLPO, but rather in the DMH/DHA (known to provide inhibitory control of the VLPO [16]), and in a range of other regions typically thought to be active during wakefulness (DMH/DHA, LH, and PVN) and arousal (amygdala, PVT, and ILT).

Thus, our c-*fos* and behavioral data both indicate an increase in anxiety and/or arousal after mRGC activation. However, previous studies have suggested that bright light has the opposite effect of inducing sleep [31, 32, 33]. To resolve this apparent contradiction, we finally tested the response of mice to bright light when challenged with the open field test. We found that in the light, wild-type (*Opn4*$^{Cre/+}$, visually intact) mice showed the same reduction in time in the center previously observed for CNO-treated hM3Dq-expressing mice (Figures 3E and 3F; total distance traveled, mean ± SEM: 1,669 ± 178 cm and 3,082 ± 245 cm in the bright and dim far-red light, respectively). This anxiogenic effect of light was recapitulated in rodless and coneless mice (*rd*^{1}*;Cnga3*$^{-/-}$; Figure 3E; total distance traveled, mean ± SEM: 2,000 ± 225 cm and 3,229 ± 271 cm in the bright and dim far-red light, respectively) but was absent in mice lacking melanopsin (*Opn4*$^{-/-}$; Figure 3F; total distance traveled, mean ± SEM: 3,286 ± 357 cm and 4,728 ± 585 cm in light and dark, respectively). The latter data are similar to those from a very recent study comparing responses to “blue” light

in wild-type and melanopsin knockout mice [34].Together, they confirm that light can have arousal and/or anxiogenic effects and that mRGCs are both sufficient and necessary for this response. The ethological significance of such divergent light responses is unclear, but the observation that the appropriate response to a sensory stimulus may be context dependent is not in itself surprising. An arousal response would have an obvious survival advantage in mice for whom exposure to bright light would invariably indicate a situation of heightened danger. More importantly, it indicates that under the right circumstances, mRGCs can drive a change in mouse behavioral state analogous to the increase in alertness and arousal experienced by humans.

SUPPLEMENTAL INFORMATION

Supplemental Information includes Supplemental Experimental Procedures, three figures, and one table and can be found with this article online at http://dx.doi.org/10.1016/j.cub.2016.06.057.

AUTHOR CONTRIBUTIONS

N.M. and R.J.L. designed the research. N.M. performed retinal and brain histology and behavioral experiments. N.M. and J.C.K. performed intravitreal injections and pupillometry. J.C.K. assisted with retinal histology. C.A.P. assisted with c-Fos immunohistochemistry and confocal microscopy. N.M. and R.J.L. wrote the manuscript with input from all authors.

ACKNOWLEDGMENTS

We thank Roger Meadows for his help with the microscopy and Samer Hattar (Johns Hopkins University) for the generous donation of the Opn4cre mouse line. We also thank Annette E. Allen for fruitful scientific discussions and her support during this work. This work was supported by grants from the European Research Council (grant 268970 to R.J.L.) and the MRC (MR/N012992/1 to R.J.L. and MC_PC_13070 Confidence in Concept award to R.J.L. and J.C.K.). The Bioimaging Facility microscopes used in this study were purchased with grants from the BBSRC, Wellcome Trust, and University of Manchester Strategic Fund. All experiments were approved by the University of Manchester Animal Welfare and Ethical Review Board.

Received: March 7, 2016
Revised: May 18, 2016
Accepted: June 24, 2016
Published: July 14, 2016

REFERENCES

1. Vandewalle, G., Maquet, P., and Dijk, D.J. (2009). Light as a modulator of cognitive brain function. Trends Cogn. Sci. *13*, 429–438.
2. Cajochen, C., Münch, M., Kobialka, S., Kräuchi, K., Steiner, R., Oelhafen, P., Orgül, S., and Wirz-Justice, A. (2005). High sensitivity of human melatonin, alertness, thermoregulation, and heart rate to short wavelength light. J. Clin. Endocrinol. Metab. *90*, 1311–1316.
3. Rahman, S.A., Flynn-Evans, E.E., Aeschbach, D., Brainard, G.C., Czeisler, C.A., and Lockley, S.W. (2014). Diurnal spectral sensitivity of the acute alerting effects of light. Sleep *37*, 271–281.
4. Lockley, S.W., Evans, E.E., Scheer, F.A., Brainard, G.C., Czeisler, C.A., and Aeschbach, D. (2006). Short-wavelength sensitivity for the direct effects of light on alertness, vigilance, and the waking electroencephalogram in humans. Sleep *29*, 161–168.
5. Viola, A.U., James, L.M., Schlangen, L.J., and Dijk, D.J. (2008). Blue-enriched white light in the workplace improves self-reported alertness, performance and sleep quality. Scand. J. Work Environ. Health *34*, 297–306.
6. Barkmann, C., Wessolowski, N., and Schulte-Markwort, M. (2012). Applicability and efficacy of variable light in schools. Physiol. Behav. *105*, 621–627.
7. Vandewalle, G., Schwartz, S., Grandjean, D., Wuillaume, C., Balteau, E., Degueldre, C., Schabus, M., Phillips, C., Luxen, A., Dijk, D.J., and Maquet, P. (2010). Spectral quality of light modulates emotional brain responses in humans. Proc. Natl. Acad. Sci. USA *107*, 19549–19554.
8. Hattar, S., Kumar, M., Park, A., Tong, P., Tung, J., Yau, K.W., and Berson, D.M. (2006). Central projections of melanopsin-expressing retinal ganglion cells in the mouse. J. Comp. Neurol. *497*, 326–349.
9. Delwig, A., Larsen, D.D., Yasumura, D., Yang, C.F., Shah, N.M., and Copenhagen, D.R. (2016). Retinofugal projections from melanopsin-expressing retinal ganglion cells revealed by intraocular injections of Cre-dependent virus. PLoS ONE *11*, e0149501.
10. Hattar, S., Lucas, R.J., Mrosovsky, N., Thompson, S., Douglas, R.H., Hankins, M.W., Lem, J., Biel, M., Hofmann, F., Foster, R.G., and Yau, K.W. (2003). Melanopsin and rod-cone photoreceptive systems account for all major accessory visual functions in mice. Nature *424*, 76–81.
11. Marc, R.E., Jones, B.W., Anderson, J.R., Kinard, K., Marshak, D.W., Wilson, J.H., Wensel, T., and Lucas, R.J. (2007). Neural reprogramming in retinal degeneration. Invest. Ophthalmol. Vis. Sci. *48*, 3364–3371.
12. Armbruster, B.N., Li, X., Pausch, M.H., Herlitze, S., and Roth, B.L. (2007). Evolving the lock to fit the key to create a family of G protein-coupled receptors potently activated by an inert ligand. Proc. Natl. Acad. Sci. USA *104*, 5163–5168.
13. Berson, D.M., Castrucci, A.M., and Provencio, I. (2010). Morphology and mosaics of melanopsin-expressing retinal ganglion cell types in mice. J. Comp. Neurol. *518*, 2405–2422.
14. Lall, G.S., Revell, V.L., Momiji, H., Al Enezi, J., Altimus, C.M., Güler, A.D., Aguilar, C., Cameron, M.A., Allender, S., Hankins, M.W., and Lucas, R.J. (2010). Distinct contributions of rod, cone, and melanopsin photoreceptors to encoding irradiance. Neuron *66*, 417–428.
15. Saper, C.B., Scammell, T.E., and Lu, J. (2005). Hypothalamic regulation of sleep and circadian rhythms. Nature *437*, 1257–1263.
16. Chou, T.C., Scammell, T.E., Gooley, J.J., Gaus, S.E., Saper, C.B., and Lu, J. (2003). Critical role of dorsomedial hypothalamic nucleus in a wide range of behavioral circadian rhythms. J. Neurosci. *23*, 10691–10702.
17. Rezai-Zadeh, K., Yu, S., Jiang, Y., Laque, A., Schwartzenburg, C., Morrison, C.D., Derbenev, A.V., Zsombok, A., and Münzberg, H. (2014). Leptin receptor neurons in the dorsomedial hypothalamus are key regulators of energy expenditure and body weight, but not food intake. Mol. Metab. *3*, 681–693.
18. Yoshida, K., Li, X., Cano, G., Lazarus, M., and Saper, C.B. (2009). Parallel preoptic pathways for thermoregulation. J. Neurosci. *29*, 11954–11964.
19. Zhang, Y., Kerman, I.A., Laque, A., Nguyen, P., Faouzi, M., Louis, G.W., Jones, J.C., Rhodes, C., and Münzberg, H. (2011). Leptin-receptor-expressing neurons in the dorsomedial hypothalamus and median preoptic area regulate sympathetic brown adipose tissue circuits. J. Neurosci. *31*, 1873–1884.
20. Aston-Jones, G., Chen, S., Zhu, Y., and Oshinsky, M.L. (2001). A neural circuit for circadian regulation of arousal. Nat. Neurosci. *4*, 732–738.
21. Kinomura, S., Larsson, J., Gulyás, B., and Roland, P.E. (1996). Activation by attention of the human reticular formation and thalamic intralaminar nuclei. Science *271*, 512–515.
22. Van der Werf, Y.D., Witter, M.P., and Groenewegen, H.J. (2002). The intralaminar and midline nuclei of the thalamus. Anatomical and functional evidence for participation in processes of arousal and awareness. Brain Res. Brain Res. Rev. *39*, 107–140.
23. Martin-Fardon, R., and Boutrel, B. (2012). Orexin/hypocretin (Orx/Hcrt) transmission and drug-seeking behavior: is the paraventricular nucleus of the thalamus (PVT) part of the drug seeking circuitry? Front. Behav. Neurosci. *6*, 75.
24. LeGates, T.A., Altimus, C.M., Wang, H., Lee, H.K., Yang, S., Zhao, H., Kirkwood, A., Weber, E.T., and Hattar, S. (2012). Aberrant light directly

impairs mood and learning through melanopsin-expressing neurons. Nature *491*, 594–598.

25. Berridge, K.C., and Kringelbach, M.L. (2015). Pleasure systems in the brain. Neuron *86*, 646–664.
26. Han, S., Tai, C., Westenbroek, R.E., Yu, F.H., Cheah, C.S., Potter, G.B., Rubenstein, J.L., Scheuer, T., de la Iglesia, H.O., and Catterall, W.A. (2012). Autistic-like behaviour in Scn1a+/- mice and rescue by enhanced GABA-mediated neurotransmission. Nature *489*, 385–390.
27. Christoffel, D.J., Golden, S.A., Heshmati, M., Graham, A., Birnbaum, S., Neve, R.L., Hodes, G.E., and Russo, S.J. (2012). Effects of inhibitor of κB kinase activity in the nucleus accumbens on emotional behavior. Neuropsychopharmacology *37*, 2615–2623.
28. Kiselycznyk, C., Hoffman, D.A., and Holmes, A. (2012). Effects of genetic deletion of the Kv4.2 voltage-gated potassium channel on murine anxiety-, fear- and stress-related behaviors. Biol. Mood Anxiety Disord. *2*, 5.
29. Bouwknecht, J.A., Spiga, F., Staub, D.R., Hale, M.W., Shekhar, A., and Lowry, C.A. (2007). Differential effects of exposure to low-light or high-light open-field on anxiety-related behaviors: relationship to c-Fos expression in serotonergic and non-serotonergic neurons in the dorsal raphe nucleus. Brain Res. Bull. *72*, 32–43.
30. Gray, J.A., and McNaughton, N. (1996). The neuropsychology of anxiety: reprise. Nebr. Symp. Motiv. *43*, 61–134.
31. Altimus, C.M., Güler, A.D., Villa, K.L., McNeill, D.S., Legates, T.A., and Hattar, S. (2008). Rods-cones and melanopsin detect light and dark to modulate sleep independent of image formation. Proc. Natl. Acad. Sci. USA *105*, 19998–20003.
32. Tsai, J.W., Hannibal, J., Hagiwara, G., Colas, D., Ruppert, E., Ruby, N.F., Heller, H.C., Franken, P., and Bourgin, P. (2009). Melanopsin as a sleep modulator: circadian gating of the direct effects of light on sleep and altered sleep homeostasis in Opn4(-/-) mice. PLoS Biol. *7*, e1000125.
33. Lupi, D., Oster, H., Thompson, S., and Foster, R.G. (2008). The acute light-induction of sleep is mediated by OPN4-based photoreception. Nat. Neurosci. *11*, 1068–1073.
34. Pilorz, V., Tam, S.K., Hughes, S., Pothecary, C.A., Jagannath, A., Hankins, M.W., Bannerman, D.M., Lightman, S.L., Vyazovskiy, V.V., Nolan, P.M., et al. (2016). Melanopsin Regulates Both Sleep-Promoting and Arousal-Promoting Responses to Light. PLoS Biol. *14*, e1002482.

Current Biology, Volume *26*

Supplemental Information

Chemogenetic Activation of Melanopsin Retinal Ganglion Cells Induces Signatures of Arousal and/or Anxiety in Mice

Nina Milosavljevic, Jasmina Cehajic-Kapetanovic, Christopher A. Procyk, and Robert J. Lucas

Figure S1 (Related to Figure 1)

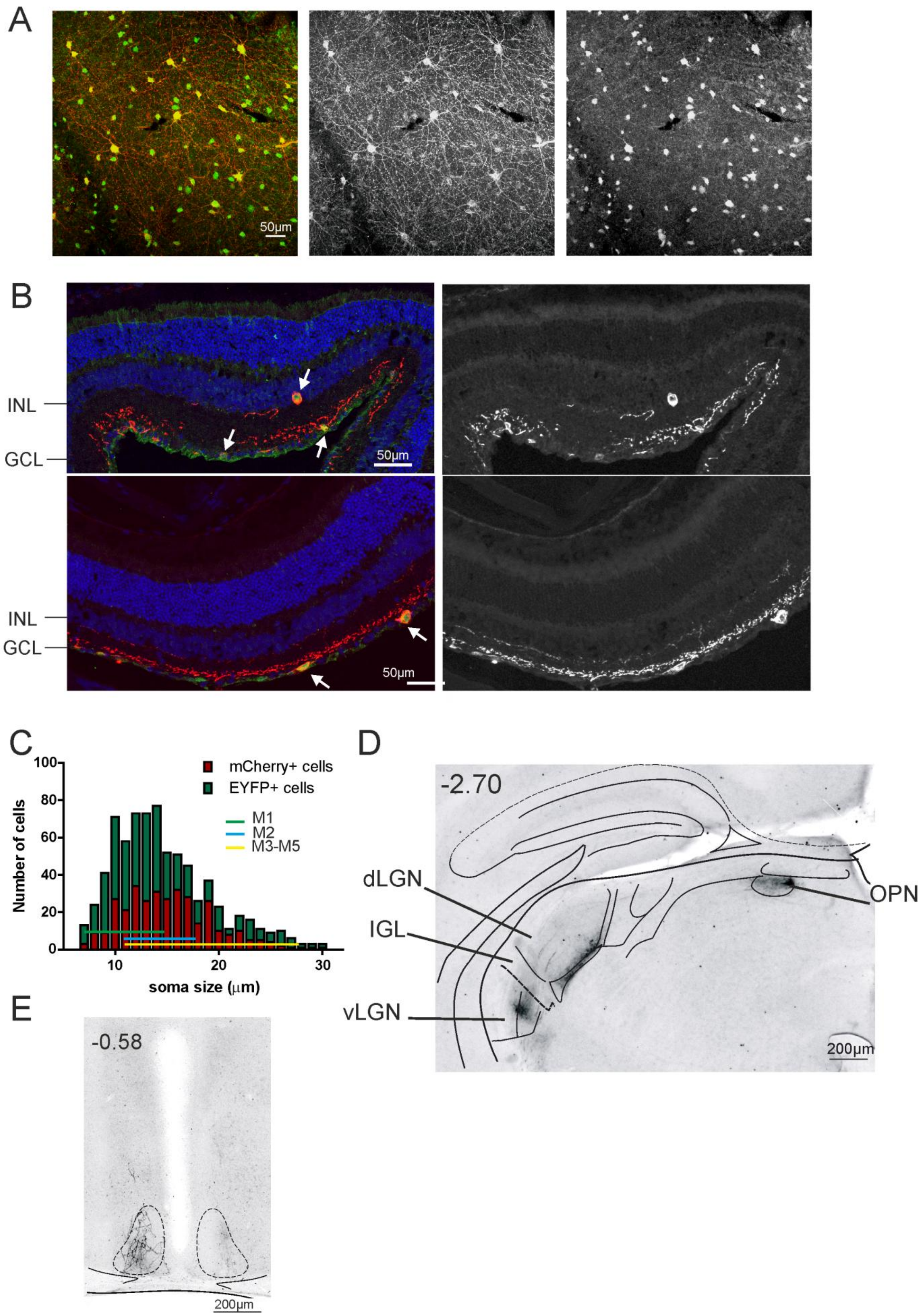

Figure S1. Related to Figure 1. Transgene expression following viral transduction of *Opn4*$^{Cre/+}$ retina. **A**) Examplar retinal wholemount from Figure 1A with monochrome versions of mCherry and GFP staining, from left to right. **B**) Examplar retinal sections from Figure 1 with monochrome versions of mCherry staining on the right. Notice the different soma sizes of transduced cells (white arrows) and a cell in INL, location of displaced M1 mRGCs [S2] and mCherry signal in OFF sublamina of IPL, DAPI stain in blue. **C**) Distribution of soma sizes of 335 mCherry+ cells and 754 EYFP+ cells sampled from 4 retinal wholemounts. Lines in different colors indicate expected range of soma sizes for different subtypes of mRGCs, green M1, blue M2 and yellow M3-M5[S1]. **D**) Coronal brain section of *Opn4*$^{Cre/+}$ mouse following injection of hM3Dq:mCherry virus in right eye shows mCherry signal (black) in axons of transduced retinal ganglion cells in oliviary pretectal nucleus (OPN), dorsal lateral geniculate nucleus (dLGN), intergeniculate leaflet (IGL) and ventral lateral geniculate nucleus (vLGN) at ~−2.70 mm from bregma. Approximate boundaries of nuclei in black from *Franklin KBJ and Paxinos G, the mouse brain, 2007*. **E**) Coronal brain section of *Opn4*$^{Cre/+}$ mouse following injection of hM3Dq-mCherry virus in right eye shows mCherry+ve processes (black) in SCN at ~−0.58 mm from bregma. The presence of mCherry processes in both SCN and OPN, and the ability of CNO to induce both pupil constriction and circadian phase shift indicates that both subtypes of M1 cells, Brn3b-positive and -negative M1 mRGCs, are trasduced[S1]. mCherry processes in other regions (along with soma size analysis) indicate M2-5 mRGC subtypes also transduced[S2].

Figure S2 (Related to Figure 1)

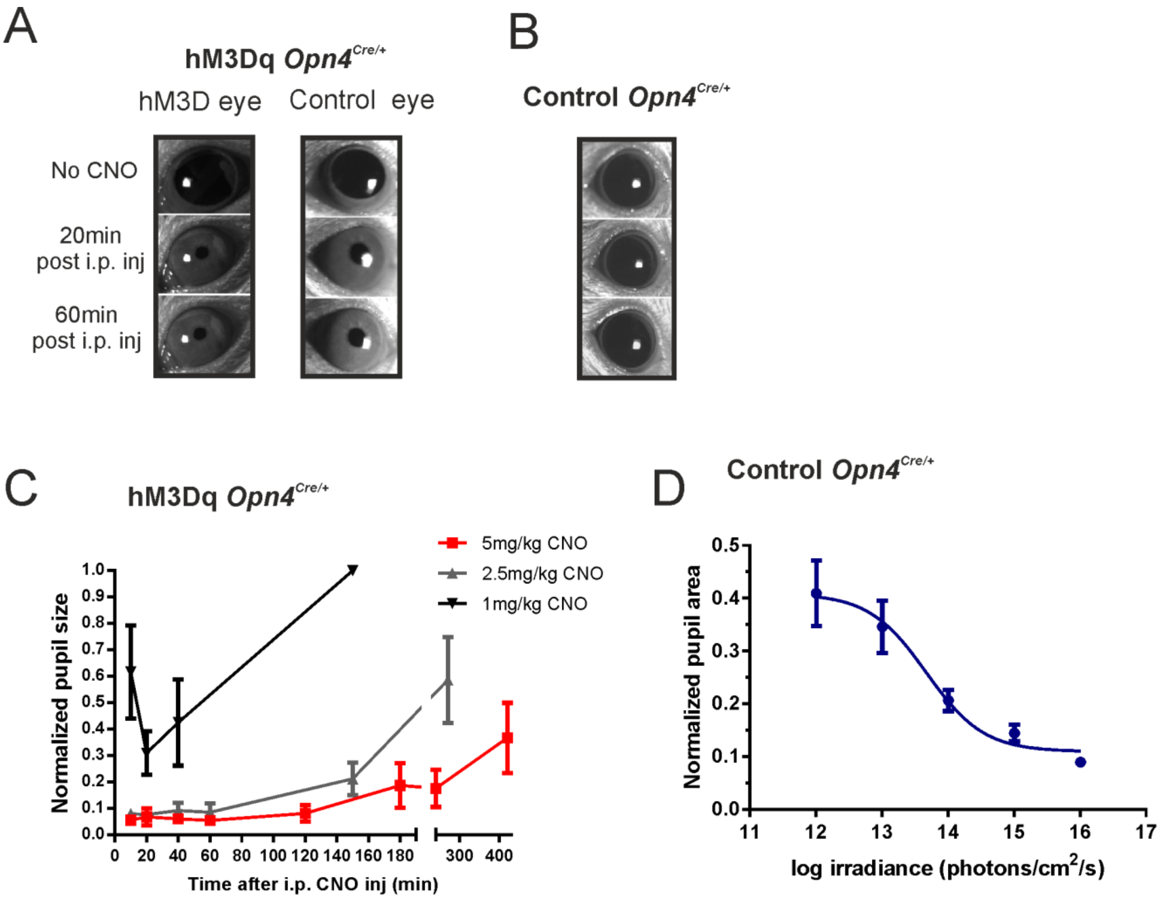

Figure S2. Related to Figure 1. Characterization of pupil response to chemogenetic activation of mRGCs. **A**) Representative frozen video images of eyes under infra-red illumination from an *Opn4*$^{Cre/+}$ mouse unilaterally expressing hM3Dq in mRGCs. Both hM3Dq and control eyes show strong constriction following CNO i.p. injection in complete darkness within first 20min that lasts for at least 120min. **B**) No change in pupil size was seen in a control *Opn4*$^{Cre/+}$ mouse treated with CNO. **C**) Mean(±SEM) pupil area (normalised to pre-CNO area=1) as a function of time after injection of CNO at 3 doses in hM3Dq-expressing *Opn4*$^{Cre/+}$ mice (n=6). **D**) Mean(±SEM) minimum normalised pupil area of control *Opn4*$^{Cre/+}$ under exposure to white light (10s) at different irradiances. Note that the pupil constriction induced by chemogenetic activation of mRGCs (C; ~90%) is equivalent to that produced by the brightest light (D), although given recent evidence that melanopsin can use a non Gq/11 phototransduction cascade [S3] the cellular mechanism may be different.

Figure S3 (Related to Figure 1)

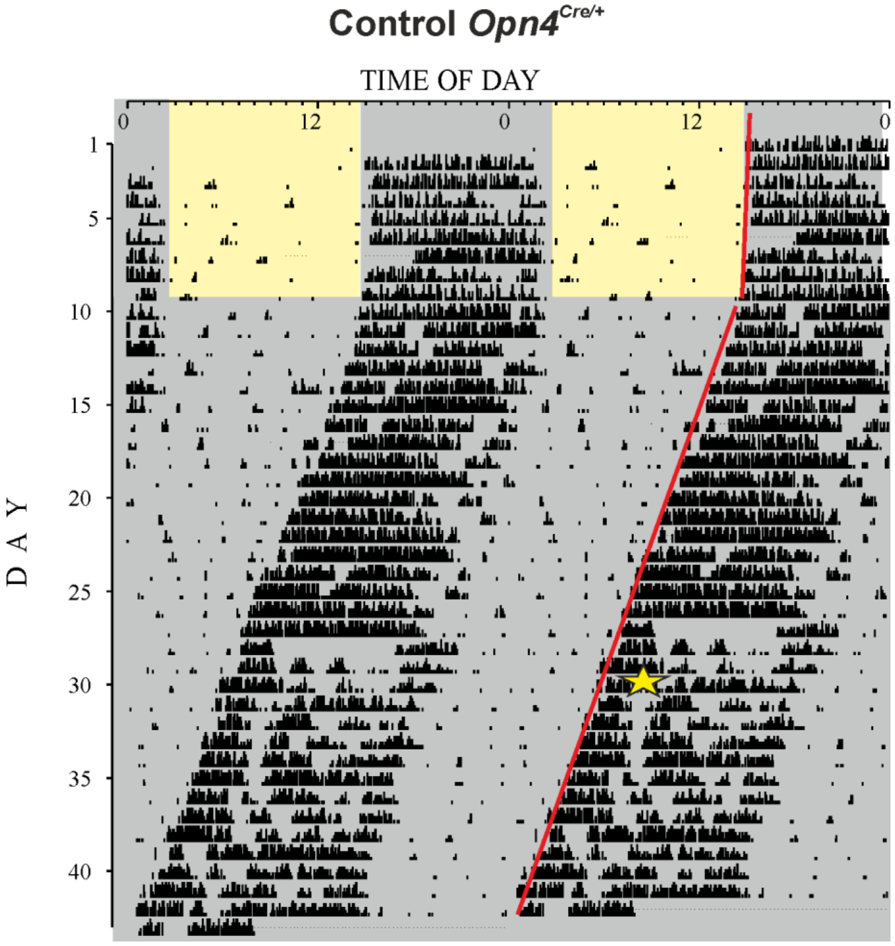

Figure S3. Related to Figure 1. Representative double-plotted actogram of wheel running activity of a control *Opn4*$^{Cre/+}$ mouse showing no change in activity to CNO administration at CT14 (asterisk designates time when a water bottle was replaced with the one containg CNO, 0.25mg/ml, for 2hrs).

Table S1. A complete statistical summary of c-Fos mapping data from Figure 2.

**Brain region**	**hM3Dq *Opn4*$^{Cre/+}$**	**Control *Opn4*$^{Cre/+}$**	**Unpaired t-test p-values**
Suprachiasmatic nuclei, SCN	1495.0 ± 220.8	618.5 ± 67.02	0.0020
Paraventricular hypothalamic nucleus, PVN	563.9 ± 39.92	334.0 ± 46.99	0.0023
Dorsomedial hypothalamus, DMH	427.4 ± 18.14	235.3 ± 23.18	0.0001
Lateral hypothalamic area, LH	209.0 ± 13.70	130.5 ± 11.42	0.0006
Ventrolateral preoptic nucleus, VLPO	331.3 ± 82.97	271.2 ± 41.77	0.5285
Basolateral amygdala, BLA	172.0 ± 31.10	88.84 ± 13.53	0.0279
Central nucleus of the amygdala, CeA	413.0 ± 91.67	165.8 ± 21.44	0.0200
Paraventricular nucleus of thalamus, PVT	521.9 ± 69.31	272.0 ± 78.93	0.0301
Intralaminar thalamic nuclei, ITL	528.8 ± 84.17	284.7 ± 59.28	0.0326
Nucleus accumbens, NAcc	229.9 ± 27.66	138.2 ± 20.76	0.0307
Lateral habenula, Hab	199.2 ± 29.73	144.3 ± 43.12	0.3193

Supplemental Experimental Procedures

Animals

Animal care was in accordance with the UK Animals, Scientific Procedures, Act (1986). Animals were kept in a 12-hour dark/light cycle at a temperature of 22ºC with food and water available *ad libitum*. Except where otherwise stated, experiments were performed on adult (4-8 months) *Opn4*$^{Cre/+}$ mice [S4]. Intravitreal injections of AAV2-hSyn-DIO-hM3Dq-mCherry vector (2.3x10^13 genomic particles/ml; The UNC Vector Core) were performed as previously reported [S5] and employed hyaluronan lyase and heparinase III (200U each) to maximise retinal penetration (total volume 2.5μl injected over 1 min). Control mice underwent the same procedure, with injections including glycosydic enzymes but with the virus replaced by vehicle (PBS). For c-Fos studies only the right eye was injected, while both eyes were injected in animals allocated to behavioural analyses. Mice were allowed at least 6 weeks to recover before being used in *in vivo* studies. Administration of Clozapine N-oxide (CNO; Abcam,) was performed via intraperitoneal (i.p.) route (5mg/kg), except where otherwise stated. The open field test was also undertaken on melanopsin knockouts (*Opn4*$^{-/-}$; [S6]) and rodless+coneless mice (*rd*^{1};*Cnga3*$^{-/-}$) produced by crossing mice carrying the *rd*^{1} mutation of the pde6b gene (C57Bl6 strain), which lesions rod phototransduction and leads to photoreceptor degeneration, with Cnga3 knockouts that lack cone phototransduction [S7].

Immunohistochemistry

Immunohistochemistry was performed as previously described [S8] on retinal wholemounts and sections fixed in methanol-free 4% paraformaldehyde. The primary antibodies used in these studies include rabbit anti-dsRed (Clontech 632496; 1:1000) and chicken anti-GFP (Abcam ab13970; 1:1000). The secondary antibodies were Alexa 488 conjugated donkey anti-chicken (Jackson Immunoresearch) and Alexa 546 conjugated donkey anti-rabbit (Life Technologies) at 1:200. Images were collected on a Leica TCS SP5 AOBS inverted confocal microscope using a 40x/0.50 Plan Fluotar objective and 1.5x confocal zoom. Brain sections were imaged with Leica M165 FC Fluorescent Stereo Microscope.

c-Fos

Mice were housed under a 12h:12h LD cycle and both hM3Dq Opn4$^{Cre/+}$ and control Opn4$^{Cre/+}$ mice were i.p. injected with CNO (5mg/kg) at CT14 under dim, deep red light (50 nW cm^{-2}, λ>650nm), after which they were placed back in the dark for a further 90 min. Then, mice were deeply anaesthetized with i.p. injection of urethane (2.2g/kg; 30%w/v; Sigma-Aldrich, UK). Once anaesthetized, the mice were perfused transcardially with 0.9% saline followed by 4% paraformaldehyde. Brains were removed, postfixed overnight in 4% paraformaldehyde, and then transferred to 30% sucrose for cryoprotection for 2 days. Coronal brain sections (50μm) were stored free floating in 0.1 M phosphate buffer. Sections were incubated in 2% hydrogen peroxidase for 20min and then in blocking buffer (0.1 M phosphate buffer, 3% Triton X-100 and 0.5% normal goat serum) for 1 h. Sections were incubated in rabbit anti-c-Fos (Calbiochem Ab-5; 1:20,000) overnight at 4°C and then visualized with a goat anti-rabbit Vectastain horseradish peroxidase kit (Vector Labs) and DAB (Vector Labs) as a chromogen. Sections were mounted on microscope slides and coverslipped with DPX. Images were acquired using the 3D Histech Pannoramic 250 Flash II slide scanner with 20x/0.80 Plan Apo objective. 3D Histech Pannoramic Viewer and ImageJ were used to count c-Fos-positive cells and measure the area of region counted from, by a blinded scorer. Between 3-5 sections per nuclei are analysed matching the same coordinates, same part of nuclei, in different animals according to Franklin KBJ and Paxinos G, the mouse brain, 2007. The number of c-Fos positive cells was normalized to the area of the region. These data were analysed using a two-tailed unpaired t-test (GraphPad Prism 6.04).

Pupillometry

Pupils were filmed in dark under infrared illumination (IR LED >900nm) by a video camera (QImaging Rolera-XR) in unanaesthetised and gently restrained mice before and after CNO injection (5 mg/kg, i.p, except where otherwise stated). Videos were analysed using ImageJ software. The areas of the pupil after CNO administration were expressed relative to the area of the pupil before CNO injection. The PLR irradiance response curve was constructed for consensual response in control *Opn4*$^{Cre/+}$ mice using a 10s full field white (150 W Metal-halide lamp; Phillips) pulse intensity controlled using neutral density filters. White light was measured using a spectroradiometer (SpectroCAL classic spectroradiometer, Cambridge Research Systems), which measured the power in milliwatt per square centimetre at wavelengths between 350 and 750 nm. Then the total photons were calculated.

Wheel-running behaviour

To assess wheel-running behaviour in LD and constant darkness, mice were individually housed in cages equipped with a running wheel (82 mm diameter, Vet-Tech, UK) under a 12h:12h LD cycle for a minimum of

10 days then released into DD for at least 14 days. The intensity of light during lights-on was 239.45 µW cm^{-2}. For phase-shifting experiments, a standard water bottle was replaced with one containing 0.25mg/ml CNO with 0.2% saccharine and 4% sucrose at CT14 for 2h. During this time, mice consumed between 650-800µl (mean±STD, 750±58µl and 700±89µl for hM3Dq and control $Opn4^{Cre/+}$ mice respectively), corresponding to a dose range of 5-6.25 mg/kg. Wheel-running data were monitored throughout the experiment and recorded in 5 min time bins using the Chronobiology Kit (Stanford Software Systems, Santa Cruz, CA, USA). These results were compared between hM3Dq and control $Opn4^{Cre/+}$ mice treated with CNO using Mann-Whitney U test, (GraphPad Prism 6.04).

Behaviour
hM3Dq $Opn4^{Cre/+}$ mice had bilateral intravitreal injections of AAV2-hM3Dq and control $Opn4^{Cre/+}$ mice had mock intravitreal injections with PBS instead of virus. Recordings were undertaken in their subjective night (between CT14 and CT17). Except where otherwise stated experiments were conducted under far red light (2.91 µW cm^{-2}, λ>680nm). Mice were injected i.p. with CNO (5mg/kg) or saline 30min before recording started. In light vs dark experiments, mice were exposed to light for 30min before the open field test experiments.

Open field
Mice were individually placed in the centre of a square arena (40 × 40 x 30cm) and allowed to explore for 10 min. Central zone is 40% of the total surface (8cm from the edge of the arena walls). Behaviour was monitored from above by a video camera. The apparatus was cleaned thoroughly between each trial. Distance travelled and time in the centre of the arena were measured using video tracking system (EthoVision XT10). These measures were compared between hM3Dq $Opn4^{Cre/+}$ mice injected with CNO with all the controls by two-way ANOVA with post hoc Bonferroni correction (GraphPad Prism 6.04). Experiments in $Opn4^{Cre/+}$, rodless+coneless (rd^1, $Cnga3^{-/-}$) and melanopsin knockout mice ($Opn4^{-/-}$) were performed under far red light (2.91 µW cm^{-2}, λ>680nm) and bright light (217 µW cm-2, halogen source).

Elevated plus maze
The apparatus consisted of two open arms (25 x 5cm) and two arms enclosed by walls (25 x 5 x 16cm) forming a cross. The arms were separated by a central platform (5 × 5 cm). The maze was elevated (52 cm). Mice were placed in the centre of the elevated plus maze facing one of the open arms that was kept consistent between mice. Behaviour was monitored from above by a video camera. The apparatus was cleaned thoroughly between each trial. Video tracking system (EthoVision XT10) was used. The time spent and number of entries in the open arms were measured as indications of risk averse behaviour. These results were compared between hM3Dq $Opn4^{Cre/+}$ mice treated with CNO and all the controls using two-way ANOVA followed by a Bonferroni post-hoc test (GraphPad Prism 6.04).

Forced swim test
Mice were individually placed in an inescapable container (35cm height, 20cm diameter) of water (25°C) filled (up to 15cm) for 6 min. Behaviour was monitored by video cameras positioned in front of the apparatus and post recording manually scored. Time spent immobile for the last 4 min of the test was calculated. Increased time spent immobile is indicative of increased depression-like behaviour. The amount of time spent immobile during the last 4 min was analysed by two-way ANOVA with post hoc Bonferroni correction (GraphPad Prism 6.04).

Supplemental References:

S1. Chen, S.K., Badea, T.C., and Hattar, S. (2011). Photoentrainment and pupillary light reflex are mediated by distinct populations of ipRGCs. Nature *476*, 92-95.

S2. Berson, D.M., Castrucci, A.M., and Provencio, I. (2010). Morphology and mosaics of melanopsin-expressing retinal ganglion cell types in mice. J Comp Neurol *518*, 2405-2422.

S3. Chew, K.S., Schmidt, T.M., Rupp, A.C., Kofuji, P., and Trimarchi, J.M. (2014). Loss of gq/11 genes does not abolish melanopsin phototransduction. PLoS One *9*, e98356.

S4. Ecker, J.L., Dumitrescu, O.N., Wong, K.Y., Alam, N.M., Chen, S.K., LeGates, T., Renna, J.M., Prusky, G.T., Berson, D.M., and Hattar, S. (2010). Melanopsin-expressing retinal ganglion-cell photoreceptors: cellular diversity and role in pattern vision. Neuron *67*, 49-60.

S5. Cehajic-Kapetanovic, J., Le Goff, M.M., Allen, A., Lucas, R.J., and Bishop, P.N. (2011). Glycosidic enzymes enhance retinal transduction following intravitreal delivery of AAV2. Mol Vis *17*, 1771-1783.

S6. Lucas, R.J., Hattar, S., Takao, M., Berson, D.M., Foster, R.G., and Yau, K.W. (2003). Diminished pupillary light reflex at high irradiances in melanopsin-knockout mice. Science *299*, 245-247.
S7. Hattar, S., Lucas, R.J., Mrosovsky, N., Thompson, S., Douglas, R.H., Hankins, M.W., Lem, J., Biel, M., Hofmann, F., Foster, R.G., et al. (2003). Melanopsin and rod-cone photoreceptive systems account for all major accessory visual functions in mice. Nature *424*, 76-81.
S8. Storchi, R., Milosavljevic, N., Eleftheriou, C.G., Martial, F.P., Orlowska-Feuer, P., Bedford, R.A., Brown, T.M., Montemurro, M.A., Petersen, R.S., and Lucas, R.J. (2015). Melanopsin-driven increases in maintained activity enhance thalamic visual response reliability across a simulated dawn. Proc Natl Acad Sci U S A.
